# SMAD9-MYCN positive feedback loop represents a unique dependency for MYCN-amplified neuroblastoma

**DOI:** 10.1186/s13046-022-02563-3

**Published:** 2022-12-20

**Authors:** Kezhe Tan, Jialin Mo, Meng Li, Yu Dong, Yujie Han, Xi Sun, Yingxuan Ma, Kai Zhu, Wei Wu, Li Lu, Jiangbin Liu, Kewen Zhao, Lei Zhang, Yujie Tang, Zhibao Lv

**Affiliations:** 1 Department of General Surgery, Shanghai Children’s Hospital, School of Medicine, Shanghai Jiao Tong University, Shanghai, China; 2 Research Center of Translational medicine, Shanghai Children’s Hospital, State Key Laboratory of Oncogenes and Related Genes, Key Laboratory of Cell Differentiation and Apoptosis of National Ministry of Education, Department of Pathophysiology, School of Medicine, Shanghai Jiao Tong University, Shanghai, China; 3 Shanghai Institute of Immunology, School of Medicine, Shanghai Jiao Tong University, Shanghai, China; 4 Department of General Surgery, Comprehensive Breast Health Center, Ruijin Hospital, School of Medicine, Shanghai Jiao Tong University, Shanghai, China; 5 Shanghai Key Laboratory of Reproductive Medicine, Department of Histoembryology, Genetics and Developmental Biology, School of Medicine, Shanghai Jiaotong University, Shanghai, China

**Keywords:** High-risk neuroblastoma, SMAD9, MYCN

## Abstract

**Background:**

Neuroblastoma (NB) is the most common extracranial solid tumor occurring during childhood and high-risk NB patients have a poor prognosis. The amplified MYCN gene serves as an important determinant of a high risk of NB.

**Methods:**

We performed an integrative screen using public NB tissue and cell line data, and identified that SMAD9 played an important role in high-risk NB. An investigation of the super-enhancers database (SEdb) and chromatin immunoprecipitation sequencing (ChIP-seq) dataset along with biological experiments of incorporating gene knockdown and CRISPR interference (CRISPRi) were performed to identify upstream regulatory mechanism of SMAD9. Gene knockdown and rescue, quantitative real-time PCR (Q-RT-PCR), cell titer Glo assays, colony formation assays, a subcutaneous xenograft model and immunohistochemistry were used to determine the functional role of SMAD9 in NB. An integrative analysis of ChIP-seq data with the validation of CRISPRi and dual-luciferase reporter assays and RNA sequencing (RNA-seq) data with Q-RT-PCR validation was conducted to analyze the downstream regulatory mechanism of SMAD9.

**Results:**

High expression of SMAD9 was specifically induced by the transcription factors including MYCN, PHOX2B, GATA3 and HAND2 at the enhancer region. Genetic suppression of SMAD9 inhibited MYCN-amplified NB cell proliferation and tumorigenicity both in vitro and in vivo. Further studies revealed that SMAD9 bound to the MYCN promoter and transcriptionally regulate MYCN expression, with MYCN reciprocally binding to the SMAD9 enhancer and transactivating SMAD9, thus forming a positive feedback loop along with the MYCN-associated cancer cell cycle.

**Conclusion:**

This study delineates that SMAD9 forms a positive transcriptional feedback loop with MYCN and represents a unique tumor-dependency for MYCN-amplified neuroblastoma.

**Supplementary Information:**

The online version contains supplementary material available at 10.1186/s13046-022-02563-3.

## Introduction

Neuroblastoma (NB) is the most common extracranial solid tumor occurring during childhood. NB threatens children’s quality of life and survival, and accounts for 8–10% of childhood cancers and 15% of cancer-related deaths in children [1, 2]. Based on age, the International Staging System (INSS), histology, genomic abnormalities, MYCN amplification and other characteristics, NB patients are classified into very low-, low-, intermediate- and high-risk groups according to the Children’s Oncology Group (COG) [3, 4]. DNA amplification of the MYCN proto-oncogene is an important determinant of a high risk in NB patients [3, 4]. Despite multimodal treatment strategies, the overall survival (OS) rate for high-risk NB patients remains below 60% [1, 5, 6].

In addition to MYCN amplification, gene abnormalities including ALK mutation [7], MYC amplification [8], TERT rearrangement [9], and ATRX inactivation [10], are risk factors for NB. However, > 25% of high-risk NB patients do not show the abovementioned genetic abnormalities [9, 11]. Moreover, NB is a specific tumor characterized by transcriptional gene abnormalities and MYCN is the most critical transcription factor (TF) [12, 13]. Therefore, more transcriptional biomarkers must be identified to improve the clinical evaluation of high-risk NB patients.

Most recent clinical trials for NB are considered “basket trials”, which are trials utilizing targeted drugs that are universally applicable for many cancer types [14, 15]. Nevertheless, neuron-derived NB is a specific type of pediatric cancer characterized by epigenetic and transcriptional aberrations [16–18]. To illustrate this uniqueness, NB is a super enhancers (SEs)-driven tumor characterized by high expression of SEs-targeted genes such as MYCN, PHOX2B, GATA3, HAND2, TBX2, ISL1, ASCL1, and LMO1 in transcriptional core regulatory circuitries (CRCs) [13, 17, 19]. Here we performed an integrative analysis of public datasets and found that SMAD9 was characterized by specific high expression and dependence among all cancer types and was positively correlated with MYCN expression and a poor prognosis in high-risk NB patients. In our models, SMAD9 promoted MYCN-amplified NB cell growth in vitro and in vivo, and its upstream and downstream targets were strongly associated with MYCN. High SMAD9 expression was associated with MYCN-associated autonomous neural tumorigenicity and a robust cancer cell cycle.

## Materials and methods

The study was approved by the Institutional Review Board (IRB) of Shanghai Children’s Hospital (SCH), Shanghai Jiao Tong University, in accordance with the principles of Declaration of Helsinki. Written informed consent to participate in this study was provided by the participants’ legal guardian or next of kin. Patients’ identities and privacy were protected and anonymized in the study. In addition, the Medical Experimental Animal Administrative Committee in Shanghai approved all animal experimental protocols.

### Patients and specimens

Patients were included based on the criteria for high-risk NB reported by the COG [3]: 1) MYCN amplification despite any other conditions; 2) INSS = 4 and age ≥ 1.5 years; and 3) INSS = 4 and the existence of genomic abnormalities. Finally, 14 high-risk NB samples obtained between January 2015 and December 2019 were selected for clinical validation. These tissue samples were excised in the operating room, immediately snap-frozen in liquid nitrogen and then stored at − 80 °C.

### Cell culture

The NB cell lines BE(2)-C, SK-N-BE2, IMR-32, SH-SY5Y and SK-N-SH were purchased from the Chinese Academy of Sciences Cell Bank. BE(2)-C, SK-N-BE2 and SH-SY5Y cells were cultured in DMEM/F12 (Gibco, #11330–032) supplemented with 10% fetal bovine serum (FBS; Sigma, #F2442) and a 1× penicillin streptomycin (P/S) solution (BasalMedia, #S110JV). IMR-32 and SK-N-SH cells were cultured in Eagle’s minimum essential medium (MEM) (BasalMedia, #L510KJ) supplemented with 10% FBS and 1 × P/S. The NB cell lines are generally categorized into 3 cell types based on morphological and biochemical properties of cells: N (neuroblastic) cells, S (substrate-adherent) cells and I (intermediate) cells. N-type cells exhibit neuronal marker enzyme activities. S-type cells contain tyrosine activity that is suggestive of stem cell capabilities. I-type cells differentially express both N-type and S-type characteristics in the transdifferentiation process between N-type and S-type cells [20]. SK-N-BE2, IMR-32 and SK-N-SH are S-type cells. SH-SY5Y are N-type cells and BE(2)-C are I-type cells [20]. Each cell line type exhibits different growth features and short tandem repeat (STR) results [20, 21]. Normal control RPE-1 fibroblasts and MCF-10 epithelial cells were cultured in DMEM/F12 supplemented with 10% FBS and 1xP/S. Virus-packaging HEK293T cells were cultured in Dulbecco’s modified Eagle medium/high glucose (DMEM) (BasalMedia, #L110KJ) supplemented with 10% FBS and 1 × P/S. STR sequencing was performed in NB cell lines by BIOWING Biotech Co., Ltd. (Shanghai, China) (Supplemental Table 1).

### Virus packaging and transfection

Lentiviral shRNA or sgRNA plasmids were constructed by inserting target oligonucleotides into pLKO.1 (Addgene, #10878) or lentiGuide-Puro (Addgene, #52963) plasmids, respectively. Plasmid DNA was extracted using a DNA extraction kit (Vazyme, DC112–01). The lentivirus was packaged by transfecting the plasmids with packaging vectors (psPAX and pMD2.G) and PEI MAX solution (Polysciences, #24765) into HEK293T cells. Afterward, the virus supernatant was collected, filtered with a 0.45 μm strainer, concentrated with PEG6000 (Sigma, #81253), resolved in PBS and then aliquoted for subsequent transfection. Cells were infected with viruses and selected for 72 hours with puromycin (0.5–2 μg/mL, Yeasen, 60210ES25), blasticidin (5–20 μg/mL, YEASEN, 60218ES60), or G418 (100–250 μg/mL, Yeasen, 60220ES03). The target oligonucleotides are listed in Supplemental Table 2.

### Immunoblotting

Cell samples were lysed in RIPA buffer (Thermo Fisher Scientific, #89900) and protein concentrations were quantified using the Pierce BCA kit (Thermo Fisher Scientific, #23225). Denatured proteins (10–20 μg/lane) were separated by sodium dodecyl sulfate polyacrylamide gel electrophoresis (SDS-PAGE) and transferred onto polyvinylidene difluoride (PVDF) membranes. Afterward, the membranes were blocked with 5% fat-free milk (BD Biosciences, #232100) in Tris buffered saline containing Tween 20 (TBST) and incubated with rabbit anti-human MYCN (1:1000; Cell Signaling Technology, #84406), mouse-anti-human Flag (1:1000; Sigma, #F1804), or rabbit-anti-human β-tubulin (1:5000; Abcam, #ab6046) primary antibodies overnight. The membranes were then incubated with HRP-conjugated goat anti-rabbit or mouse IgG (0.2 μg/ml; Pierce, #31460 or #31430). A luminescent image analyzer (Fujifilm, LAS-4000) was used to visualize the bands after an incubation with enhanced chemiluminescence reagents (Millipore, WBKLS0500).

### Quantitative reverse transcription PCR (Q-RT-PCR)

Approximately 25 mg tissue samples or 2.5 × 10^5^ cells were lysed in TRIzol reagent (Thermo Fisher Scientific, #TR118) for total RNA extraction. RNA was reverse transcribed to cDNA with a High-Capacity RNA-to-cDNA kit (Thermo Fisher Scientific, #4387406). Quantitative PCR was performed using an Applied Biosystems QuantStudio™ 5 Real-Time PCR System (Thermo Fisher Scientific, #A34322) with SYBR Green Master buffer (ROX) (Thermo Fisher Scientific, #A25742). GAPDH was used as an internal control, and mRNA levels were calculated with the 2^delta Ct approach. The primer sequences are summarized in Supplemental Table 3.

### Cell viability assay

Cells were plated in 96-well plates in triplicate at a density of 750 cells/well (small cells except for SK-N-SH and RPE-1 cells) or 375 cells/well (large cells) in 100 μL of culture medium. Then the CellTiter-Glo® luminescent cell viability assay (Promega, #G7573) was performed to assess cell viability at days 0, 2, 4 and 6 according to the manufacturer’s protocol.

### Clone formation assay

A total of 375 cells/well (small cells) or 187 cells/well (large cells) were seeded in 12-well plates. Fresh medium was added every 5 days in the first 7 days. Half of the medium was removed, and the same amount of fresh medium was added every 4 days afterward. Generally, colonies were visible after 8–21 days, and the cells were washed with PBS, fixed with a 10% neutralized formaldehyde solution, and then stained with 0.5% crystal violet (Sigma, #C6158-100G) containing 25% methanol.

### SMAD9 constructs

The sequence of the SMAD9 coding sequence (CDS) was screened to design a spot mutation without a change in amino acid sequence in the shSMAD9 target site. Wild-type (WT) and mutated (MUT) SMAD9 CDSs were cloned into the G418-resistant vector plenti-EF1α-MCS-IRES-Neo-WPRE (EcoRI and BamHI) by homologous recombination using the ClonExpress kit (Vazyme, #C112). The empty vector (EV), the rebuilt SMAD9-WT and SMAD9-MUT plasmids were applied for virus packaging in HEK293T cells and NB stably transfected cells (STCs) were established with G418 selection. Thereafter, STCs were transfected with shSMAD9 and selected with puromycin.

### NB patient-derived Xenograft (PDX) and cells (PDCs)

Two primary NB samples from high-risk patients (SCH-NB010# and SCH-NB016#) were orthotopically xenografted into NSG mice in the first passage. Then, xenografts in the second, third and fourth passages in vivo were subcutaneously transplanted (passaged) into NSG mice. PDCs were extracted in the fourth passage and cultured in DMEM/F12 supplemented with 10% FBS. According to previous reports [22, 23], the formation of neuron-shaped cells indicated good cell line generation. After the formation of large numbers of neuron-like cells, NB PDCs were digested and transfected with shSMAD9 virus for further analysis.

### Tetracycline-inducible SMAD9 knockdown (Tet-on)

The shSMAD9 sequences were cloned into the Tet-on puromycin-resistant plasmid (Addgene, **#**21915). Empty vector (EV) and Tet-on-shSMAD9 plasmids were applied for virus packaging in HEK293T cells and for NB STC establishment with puromycin selection. Doxycycline (Dox) was used in Tet-on STCs. Therefore, STCs were expanded for validation in vitro (1 μg/mL Dox to induce SMAD9 knockdown) and tumor growth in vivo (2 mg/mL Dox with 2% sucrose).

### Animal experiments

BALB/c nude female mice (4–6 weeks) were purchased from the Experimental Animal Center of the Chinese Academy of Sciences (Shanghai, China). For subcutaneous cell line xenografts, 5 × 10^6^ NB Tet-on STCs were subcutaneously transplanted in the dorsal flanks of mice on each side. When the tumors reached the volume of approximately 100 mm^3^, the mice were randomly divided into two groups and provided water containing 2 mg/mL Dox with 2% sucrose or 2% sucrose as a control. Tumor volumes were measured every 3 days with the formula 1/2 (long axis * short axis^2). Mice with tumors > 1500 mm^3^ were euthanized.

### Histological analysis

Histological analyses, including hematoxylin-eosin (HE) and immunohistochemical (IHC) staining, were performed by Servicebio Biotechnology Company (Shanghai, China). IHC staining was performed using primary antibodies against Ki-67 (Servicebio, #GB121499) and cleaved caspase-3 (Cell Signaling Technology, #9661). The stained cells were counted as the percentage of total positive cells in the five random fields of view using the IHC profiler plugin in the ImageJ software (v1.52p, USA).

### ChIP-seq and ChIP-qPCR analysis

The Flag-tagged SMAD9 CDS was reconstructed in the plasmid pCDH-CMV-MCS-EF1-Puro using the ClonExpress kit (Vazyme, #C112). Empty vector (EV) and Flag-tagged SMAD9 CDS plasmids were applied for virus packaging in HEK293T cells and NB STC establishment with puromycin selection. Thereafter, NB STCs (1 × 10^7^) were digested with micrococcal nuclease (New England Biolabs, #M0247S) and sonicated for ChIP experiments. Briefly, 10 μg of chromatin were immunoprecipitated with a Flag antibody (Cell Signaling Technology, #14793S), or H3K27Ac antibody (AM39133, Active Motif) and 50 μL Pierce ChIP-grade Protein A/G Magnetic Beads (Thermo Scientific, #26162). Immunoprecipitated DNA and INPUT DNA were then purified and sequenced by Romics (Shanghai, China). ChIP-seq data were first mapped against the human genome build hg19 using BowTie2 [24]. Then, a model-based analysis of ChIP-seq data (MACS2: v2.2.7.1) was used to identify peak regions of ChIP-seq enrichment [25]. Peaks were annotated by performing a HOMER analysis [26]. DeepTools was used to generate BigWig files for visualization [27]. ChIP-seq data from biological experiments and public datasets were subjected to the same pipeline.

For the ChIP-qPCR analysis, 5–10 × 10^6^ cells were harvested and 5–10 μg of chromatin were immunoprecipitated with a Flag antibody and 50 μL of Pierce™ ChIP-grade Protein A/G Magnetic Beads. qPCR was performed using SYBR Green buffer (Thermo Fisher Scientific, #A25742). The primers were designed according to the peak sequence of Flag on the promoter of MYCN (Supplemental Table 4).

### CRISPR interference (CRISPRi) assay

Human MYCN-amplified BE(2)-C, SK-N-BE2 and IMR-32 NB cells were transduced with lenti-dCas9-KRAB-blast (Addgene, #89567) to generate dCas9-expressing STCs, and then lentiviral sgRNA was transduced into dCas9 expressing cells. Forty-eight hours after infection with the lentiviral sgRNA, positive cells were selected with puromycin for 3–5 days. Then, the screened cells were harvested for Q-RT-PCR, immunoblotting, cell viability and colony formation assays. The sgRNA target oligonucleotides were designed using online software (https://www.benchling.com/) according to the promoters or enhancers of target genes (Supplemental Table 5).

### Luciferase reporter assay

A luciferase reporter assay was performed as described in a previous study [28]. The MYCN promoter or SMAD9 enhancer sequences were cloned into pGL3-luc vectors according to the peak sequences of SMAD9-Flag to MYCN or MYCN to SMAD9 based on ChIP-seq data. The cloning PCR primers are presented in (Supplementary Table 6).

For MYCN knockdown, we constructed BE(2)-C Tet-on shMYCN cells by infecting wild-type BE(2)-C cells with the pLKO-Tet-On-shMYCN virus followed by continuous puromycin selection. Then, BE(2)-C Tet-on shMYCN cells were transfected with pGL3-luc based reporter plasmids and Renilla pGL3-Rluc control plasmid (ratio, 10:1). After 24 hours, transfection media were replaced with media containing or lacking doxycycline (1 μg/ml), and cells were harvested 48 h later. Procedures similar to those described above were used for BE(2)-C Tet-on shSMAD9 cells. Samples were assayed with a dual luciferase assay system (Promega, E1910). Firefly luciferase activity was normalized to Renilla luciferase activity.

### Public data acquisition

The NB tissue datasets (GSE12460, GSE13136, GSE16476, GSE49710 and GSE79910), NB cell line datasets (GSE28019, GSE80397, GSE121529 and GSE124451), NB xenograft dataset (GSE90121), normal adrenal gland datasets (GSE3526, GSE7307 and GSE8514), normal neural crest cell line dataset (GSE14340) and ChIP-seq dataset (GSE94822) were downloaded from the Gene Expression Omnibus (GEO) database (https://www.ncbi.nlm.nih.gov/geo/). The NB tissue RNA-seq dataset EGAS00001001308 (abbreviated as EGAS) and microarray dataset TARGET were obtained from the R2 database (https://hgserver1.amc.nl/cgi-bin/r2/main.cgi). The noncancerous cell line datasets included epithelium-derived MCF-10A cells (GSE17785), epidermal keratinocytes (GSE7216) and endometrial cells (GSE16906) from the R2 database. Data on expression and dependency in cancer cell lines were downloaded from the DepMap database (https://depmap.org/portal/; version 22Q1). ChIP-seq data for SEs were downloaded from the super-enhancer database (SEdb; http://www.licpathway.net/sedb/) as described in the previous reports [29].

### Analysis of RNA sequencing data

For the RNA sequencing analysis of our own samples, trim galore was used to automatically detect and trim adaptors. Reads were mapped to the hg19 reference genome using HISAT2. Read counts were generated using HTSeq (version 0.11.1) and fragments per kilobase million (FPKM) values were calculated from the number of reads that mapped to each gene sequence, and the gene length and sequencing depth were considered.

### Identification of differentially expressed genes (DEGs) and construction of a Venn diagram

The DEGs identified among NB tissues vs. normal adrenal glands, NB cell lines vs. neural crest cells and shScramble vs. shSMAD9 MYCN-amplified cells were analyzed using the false discovery rate (FDR) moderated limma test (package “DESeq2” in R). The cutoff was set for DEG selection based on the criterion |log_2_ (fold change, FC)| > 1 with *P* < 0.05 in public datasets and |log_2_FC| > 0.4 with *P* < 0.05 in shSMAD9 RNA-seq data. A Venn diagram was constructed to visualize the overlapping genes using an available online tool (http://bioinformatics.psb.ugent.be/webtools/Venn/).

### Gene Set Enrichment Analysis (GSEA)

GSEA was performed with GSEA 4.1.0 software according to the online instructions (http://www.broadinstitute.org/gsea/index.jsp). All gene sets were downloaded from the official GSEA website, which includes 50 hallmark gene sets (v7.4) and MYCN-associated gene sets: WEI_MYCN_TARGETS_WITH_E_BOX, NMYC_1, KIM_MYCN_AMPLIFICATION_TARGETS_UP and LASTOWSKA_COAMPLIFIED_WITH_MYCN. In addition, we extracted the top 500 genes (ranked by *P* value) after MYCN knockdown (KD) from GSE80397 and GSE121529 and built the gene sets MYCN_TARGETS_GSE80397 and MYCN_TARGETS_GSE121529. An FDR q-value < 0.25 was considered statistically significant.

### Protein-protein interaction (PPI) network, Cytoscape and Gene Ontology (GO)/Kyoto Encyclopedia of Genes and Genomes (KEGG) analyses

The STRING (http://string-db.org/) database was applied to determine the PPI network of 784 common downregulated genes with an interaction score of 0.4, after which Cytoscape combined with the CytoHubba plugin was used to visualize the PPI network and hub genes. GO/KEGG enrichment analyses were performed using the “clusterProfiler” and “org.Hs.eg.db” packages in R, and the top items were visualized.

### Statistical analyses

GraphPad Prism v9.2.0 or R v3.6.3 software was applied for statistical analysis. Comparisons between two groups were performed using unpaired two-tailed Student’s *t* test, and comparisons among more than two groups were performed using one-way ANOVA. Tumorigenicity in vivo was compared between the two groups using two-way ANOVA. The log-rank (Mantel Cox) test was used to analyze the Kaplan-Meier survival curves. *P* < 0.05 was considered statistically significant.

## Results

### High SMAD9 expression is an indicator of a poor prognosis for a subset of high-risk patients with NB

**Fig. 1:**
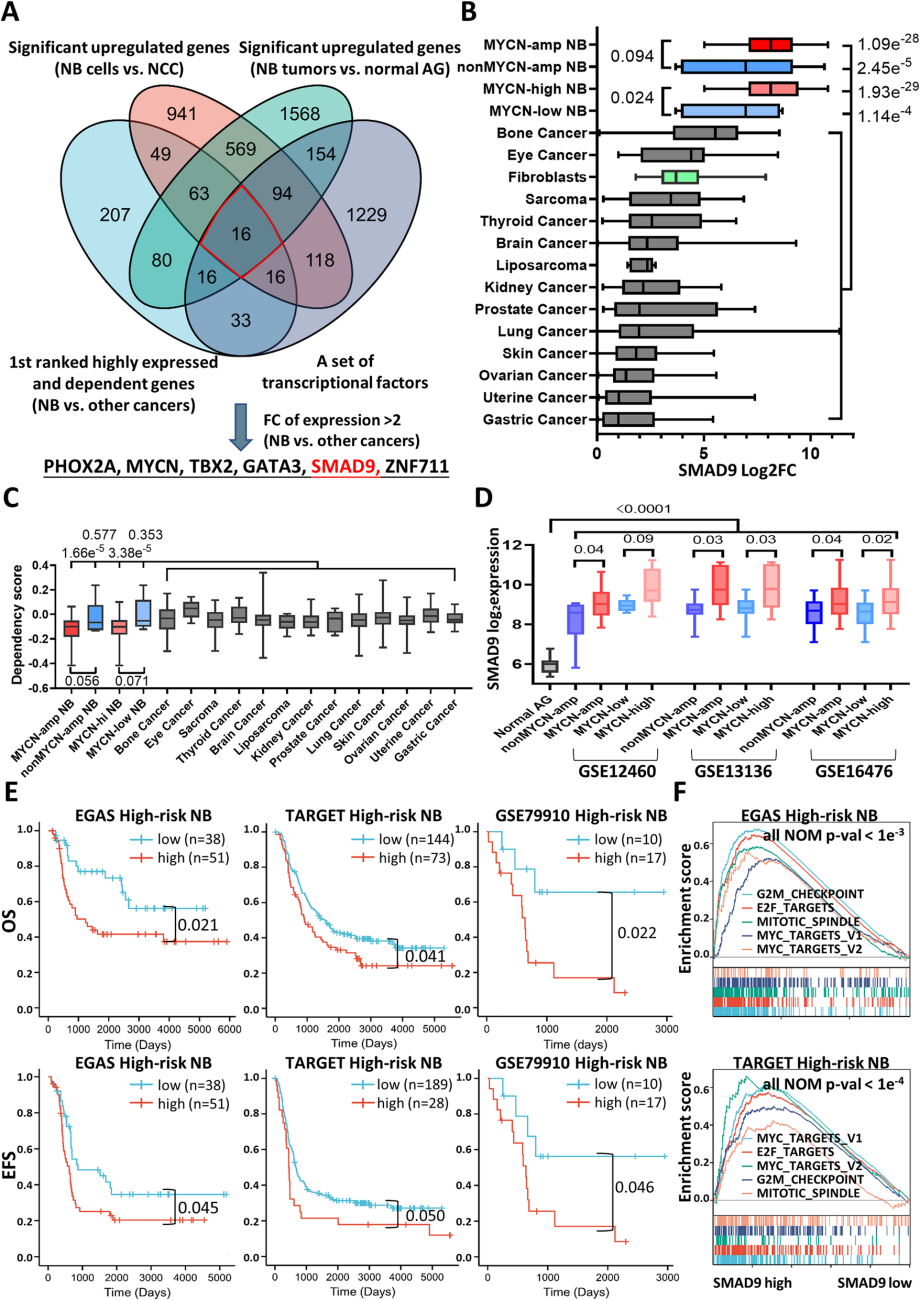
High SMAD9 expression is an indicator of a poor prognosis of high-risk NBs. **A** Venn diagram showing the 16 overlapping NB-specific transcripts compared with normal and other cancer cells or tissues. **B** Transcriptional profile of SMAD9 among the top 15 cancer cell lines in the DepMap database, where the median expression in gastric cancer normalized to 1, along with SMAD9 the expression profile in MYCN-amp NB and nonMYCN-amp NB, as well as MYCN-high NB and MYCN-low NB (based on the average MYCN transcript level). **C** Dependency score of SMAD9 among different subtypes of NB and 14 other cancer cell lines in the DepMap portal. **D** SMAD9 expression profile in normal and high-risk NB tissues in the GPL570 microarray platform. The MYCN-high and MYCN-low groups were separated by the median expression of MYCN. **E** Kaplan-Meier curves of the SMAD9-high group and SMAD9-low group from high-risk NB patients among the 3 datasets. **F** Representative “Hallmark” molecular signatures revealed by GSEA in 2 high-risk NB cohorts and their NOM *p* values. AG: adrenal gland; CCLE: cancer cell encyclopedia; EFS: event-free survival; EGAS: dataset EGAS00001001308; FC: fold change; GSEA: gene set enrichment analysis; MYCN-amp: MYCN amplification; NB: neuroblastoma; NCC: neural crest cells; NOM: normalized; nonMYCN-amp: non-MYCN amplification; OS: overall survival; TARGET: therapeutically applicable research to generate effective treatments

The workflow of the whole study is shown in Fig. S1. An integrative analysis was performed by utilizing a DepMap dataset of the 1st ranked highly expressed and dependent genes (Figs. S2A and S2B) and public high-risk NB tissue and cell line datasets (Figs. 1A and S2A) to identify transcripts that were specifically expressed in high-risk patients with NB. We identified 16 potential NB risk-related genes (Fig. 1A) and ranked them by fold change in expression (NB compared with other cancer types; Fig. S2C). Notably, we found that SMAD9 expression levels were more than 6-fold higher in NB, similar to the well-known transcripts including PHOX2A, MYCN, TBX2 and GATA3, indicating specificity (Figs. 1A and S2C).

In NB, SMAD9 exhibited high expression among all cancer and normal cells (Figs. 1B and S2D). Moreover, SMAD9 expression was positively associated with high expression of MYCN and the MYCN amplification status in cell lines (Figs. 1B and S2D). An analysis of public functional genomics data showed that NB tumor viability depended on SMAD9, and its dependency score was potentially correlated with the MYCN amplification status and MYCN expression level (Fig. 1C). In addition, we detected a higher SMAD9 expression in high-risk NB tissues compared with normal adrenal gland (AG) tissues, and SMAD9 expression was higher in MYCN-amp and MYCN-high NB tumors (Fig. 1D). Interestingly, low-stage or nonhigh-risk NB tumors exhibited relatively higher expression of SMAD9 (Fig. S2E), therefore, the correlation between SMAD9 and MYCN expression was significantly diminished in nonhigh-risk NB (Fig. S2F) and all NB tumors (Fig. S2G).

We selected several public datasets and collected clinical information from high-risk NB patients in Shanghai Children’s Hospital (SCH) to further analyze the prognostic significance of SMAD9 (Fig. S3A). High SMAD9 expression indicated a poor prognosis for patients in the high-risk cohorts (Figs. 1E and S3B-S3C). Similar to the results of SMAD9 expression in NB tumors (Figs. 1D and S2E-S2G), SMAD9 was not an indicator of inferior outcome in nonhigh-risk NB (Fig. S3D) and all NB patients (Fig. S3E). MYCN-amp NB was a subset of high-risk NB, and high SMAD9 expression also indicated a poor prognosis for MYCN-amp NB patients (Figs. S3C and S3F). Notably, high-risk NB tissue samples with high expression of SMAD9 were enriched in gene sets of GSEA concerning oncogenic malignancy terms (Fig. 1F). Taken together, our integrative analyses indicate that SMAD9 is expressed at high levels in NB cell lines and tissues and is associated with a poor prognosis of high-risk patients.

### SEs-targeted SMAD9 is potentially activated by NB-specific genes in CRC

**Fig. 2:**
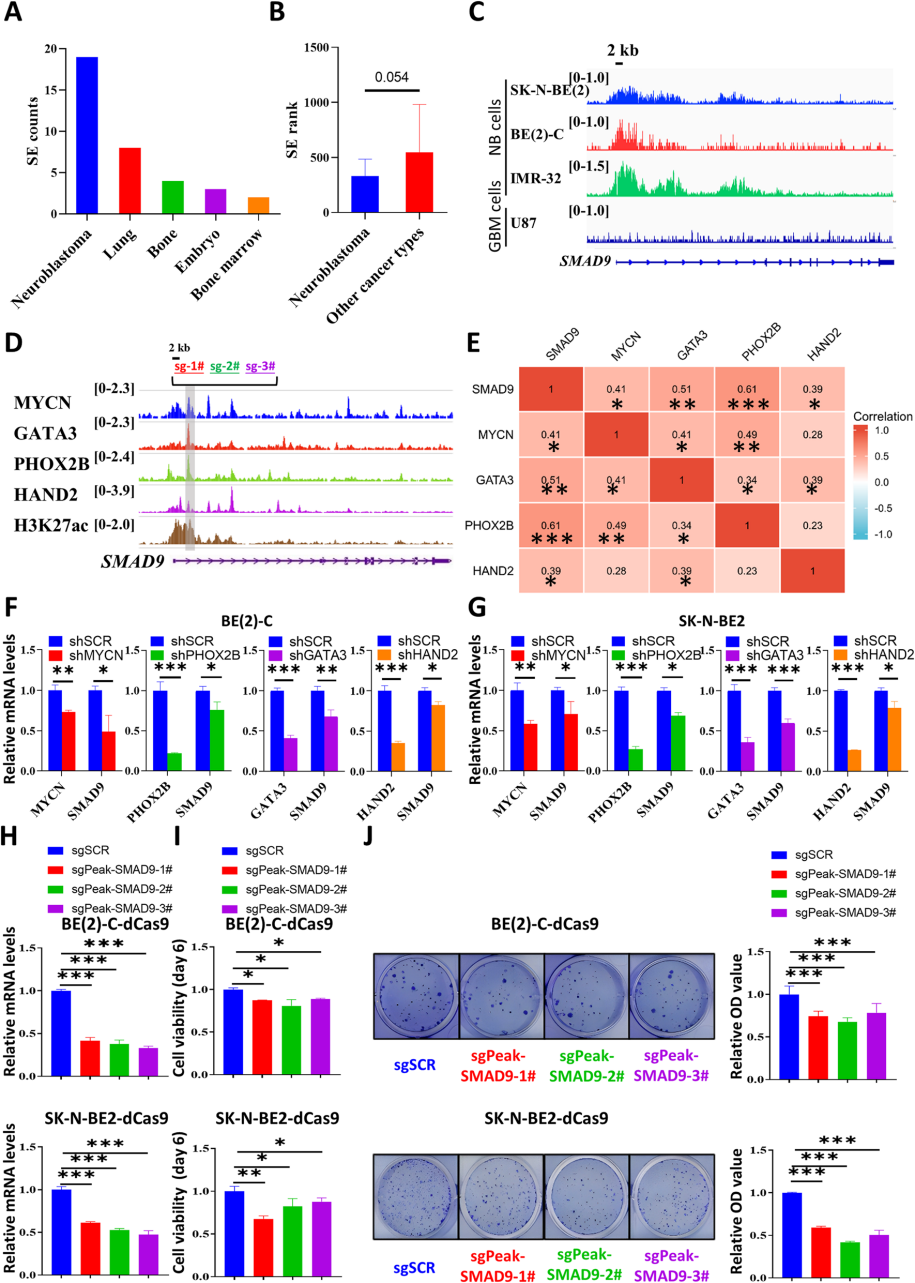
NB-specific genes in CRC potentially induce high expression of SMAD9. **A, B** SEdb analysis showing the highest number of SE counts in NB (**A**) and other cancer types described above in terms of rank (**B**). **C** Gene track showing NB-specific binding signals in the SMAD9 SEs region based on SEdb. **D** Gene track showing high binding signals for TFs in CRC as well as H3K27ac in the SMAD9 enhancer region. Sg-1#, 2# and 3# indicate the sgRNA primers based on the locations for subsequent CRISPRi experiments. **E** Matrix showing the gene-gene correlation value among MYCN, GATA3, PHOX2B, HAND2 and SMAD9 expression in NB cells in the DepMap portal. **F, G** Q-RT-PCR analyses of TF genes in CRC and SMAD9 after knockdown of one of these TFs in BE(2)-C (**F**) and SK-N-BE2 (**G**) cells. **H** Q-RT-PCR analyses of SMAD9 with disrupted binding of the CRC enhancer region to SMAD9 using the CRISPRi system in MYCN-amp dCas9 STCs. **I, J** Cell viability on day 6 (**I**), colony formation (J; left panel) and quantification (J; right panel) of MYCN-amp dCas9 STCs with disrupted binding of the CRC enhancer region to SMAD9. CRC: core regulatory circuit; dCas9: dead Cas9; GBM: glioblastoma multiforme; MYCN-amp: MYCN amplification; NB: neuroblastoma; SEs: super-enhancers; SEdb: super-enhancers database; shSCR: shRNA scrambled control; sgSCR: sgRNA scrambled control; STCs: stably transfected cells. *P < 0.05, **P < 0.01 and *** *P* < 0.001

We performed an upstream regulatory analysis in SEdb to investigate the reason why SMAD9 was highly and specifically expressed in NB, showing that SMAD9 was a specific SEs-targeted gene in NB compared to other cancer types according to SE counts (Figs. 2A and S4A-B), SE rank (Fig. 2B) and SE binding patterns (Fig. 2C). In addition, NB is a peripheral nervous tumor with high specificity driven by SE-targeted genes in CRC [13, 17]. Therefore, we analyzed public ChIP-seq data (GSE94822) and found that MYCN, GATA3, PHOX2B and HAND2 (TFs in CRC) shared peaks with H3K27ac peaks at the enhancer region of SMAD9 in MYCN-amplified BE(2)-C cells (Fig. 2D). Data from the DepMap portal showed that SMAD9 expression was positively correlated with these SE-targeted genes (Fig. 2E).

We investigated some knockdown datasets to validate the binding potency of these SEs-targeted genes on SMAD9 enhancer region and found that SMAD9 expression was downregulated by knocking down MYCN (GSE121529) and PHOX2B (GSE124451) (Fig. S4C). In addition, we used shRNAs to knockdown MYCN, GATA3, PHOX2B and HAND2. Interference with one of these 4 genes decreased SMAD9 mRNA levels in BE(2)-C cells (Fig. 2F) and SK-N-BE2 cells (Fig. 2G). Moreover, the CRISPRi assay showed that the interruption of the binding of CRC signals to SMAD9 reduced SMAD9 expression in NB cells (Fig. 2H). The disturbance of the binding sites of the CRC signal to SMAD9 reduced NB cell viability (Fig. 2I), and colony formation (Fig. 2J). Taken together, these data suggest that NB-specific TFs in CRC might bind the SMAD9 super-enhancers region and induce high SMAD9 expression in NB.

### SMAD9 knockdown suppresses MYCN-amplified NB growth

We detected MYCN expression in NB cell lines (SK-N-SH, SH-SY5Y, SK-N-BE2, BE(2)-C, IMR-32) at the protein level (Fig. S5A) and SMAD9 and MYCN mRNA expression in NB cell lines, a fibroblast cell line (RPE-1), epithelium-derived cell line (MCF-10) and NB tissues from SCH (Fig. S5B). We did not observe a significant difference in SMAD9 expression between MYCN-nonamplified and MYCN-amplified cell lines and tissues, but MYCN-high NB tissues (classified by the median expression) had a significantly higher SMAD9 level than MYCN-low NB tissues (Fig. S5B). In addition, we analyzed SMAD9 expression in noncancerous cells using Q-RT-PCR (Fig. S5B) and in datasets (Fig. S2D and S5C), and observed lower expression of SMAD9 in noncancerous cells.

**Fig. 3:**
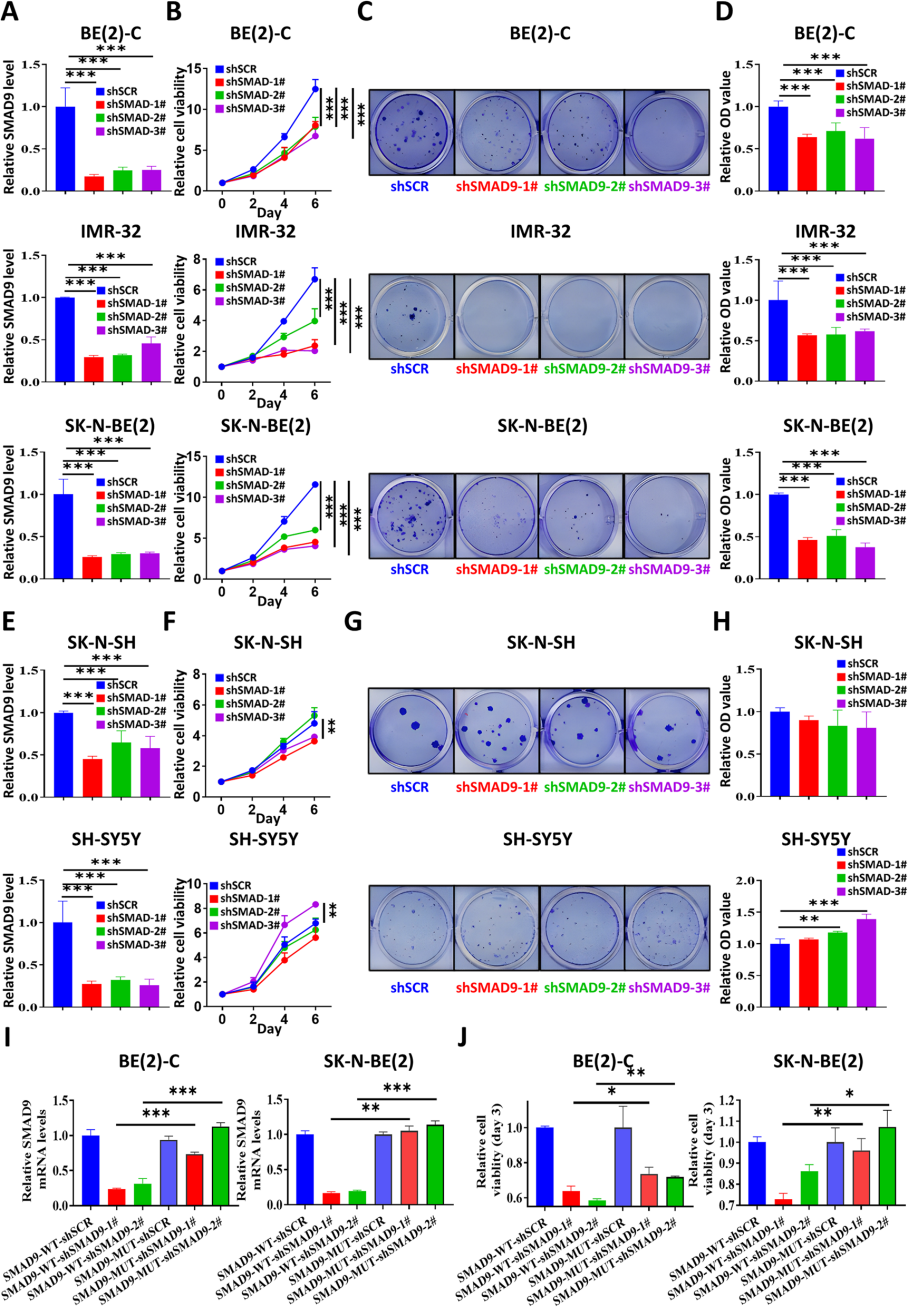
SMAD9 knockdown suppresses MYCN-amplified NB growth. **A** SMAD9 knockdown in MYCN-amp cells (BE2-C, IMR-32 and SK-N-BE2), as measured using Q-RT-PCR. **B** The viability of MYCN-amp cells was measured using CTG after knockdown of SMAD9. **C, D** Colony formation of MYCN-amp cells after SMAD9 knockdown (**C**) and quantification of CVS results (**D**). **E, F, G, H** SMAD9 knockdown in non-MYCN-amp cells (SK-N-SH and SH-SY5Y in E) measured using Q-RT-PCR (**E**) and cell viability (**F**) and colony formation (**G, H**) after SMAD9 knockdown. **I, J** Exogenous SMAD9 overexpression without (WT) or with (MUT) SMAD9-CDS mutation and shSMAD9 transfection. SMAD9 mRNA levels in MYCN-amp cells measured using Q-RT-PCR (**I**) and cell viability after SMAD9 overexpression (**J**). CDS: coding sequence; CTG: Cell-Titer-Glo; CVS: crystal violet staining; MYCN-amp: MYCN amplification; MUT: mutation; non-MYCN-amp: non-MYCN amplification; Q-RT-PCR: quantitative reverse transcription polymerase chain reaction; shRNA: short hairpin RNA; shSCR: shRNA scrambled control; WT: wild type. * *P* < 0.05, **P < 0.01 and *** *P* < 0.001

Next, we used shRNAs to knock down SMAD9 expression in MYCN-amplified cells (BE2C, IMR32 and SK-N-BE2 cells; Fig. 3A), and SMAD9 knockdown significantly inhibited NB growth in vitro (Fig. 3B)**.** In addition, a colony formation assay indicated that SMAD9 inhibition suppressed MYCN-amplified NB growth (Fig. 3C and D). However, SMAD9 knockdown in MYCN nonamplified cells (SK-N-SH and SH-SY5Y cells; Fig. 3E) did not significantly repress NB growth in vitro (Fig. 3F) or even promote NB growth (lower panel; SH-SY5Y cells in Fig. 3F). Similarly, SMAD9 knockdown in MYCN nonamplified cells did not inhibit colony formation (Fig. 3G and H). These results were consistent with functional genomics data in the DepMap portal (Fig. S5D). Moreover, we generated two types of MYCN-amplified patient derived cells (PDCs named SCH-NB010# and SCH-NB016#) and found that knockdown of SMAD9 suppressed NB growth (Figs. S5E and S5F). Finally, reconstitution of SMAD9 expression (Fig. 3I) increased the viability of MYCN-amplified NB cell lines (Fig. 3J). Based on these results, SMAD9 knockdown inhibits MYCN-amplified NB growth in vitro.

**Fig. 4:**
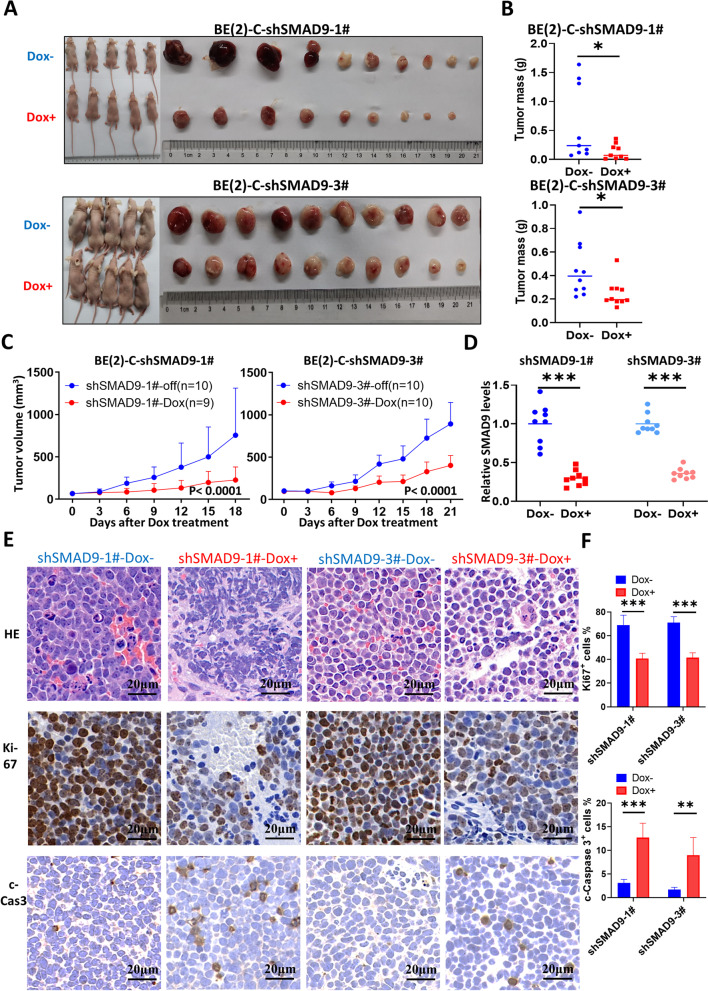
SMAD9 knockdown inhibits NB growth in vivo. **A** Photographs of nude mice subcutaneously xenografted with BE(2)-C cells that had been stably transfected with Tet-on-shSMAD9 constructs. **B, C** Tumor mass (**B**) and tumor volume growth curve (**C**) in xenografted mice. **D** SMAD9 knockdown in shSMAD9–1# and shSMAD9–3# of Dox-off and Dox-on mice. **E** Representative images of HE and IHC (Ki-67 for proliferation and c-Cas3 for apoptosis) staining (40x magnification) in tumor tissue from shSMAD9 Dox-off and Dox-on mice. **F** Quantification of the percentage of Ki-67 and c-Cas3 positive cells. c-Cas3: cleaved caspase 3; Dox: doxycycline; HE: hematoxylin and eosin; IHC: immunohistochemistry. * *P* < 0.05, **P < 0.01, and *** *P* < 0.001

We employed Tet-on shRNA methods to induce SMAD9 knockdown in BE(2)-C Tet-on-shSMAD9 STCs as a method to validate the function of SMAD9 in MYCN-amplified NB in vivo (Fig. S6A), and found that doxycycline-induced SMAD9 knockdown suppressed NB growth in vitro **(**Figs. S6B-S6D). Afterward, we suppressed SMAD9 expression in vivo and found that SMAD9 knockdown in NB xenografts led to a decreased tumor volume and mass (Fig. 4A-C). SMAD9 knockdown was validated using a Q-RT-PCR assay **(**Fig. 4D**)**. The histological analysis indicated that SMAD9 knockdown abolished the tumor microstructure (Figs. 4E and S6E), decreased cell proliferation and increased apoptosis (Figs. 4E, F and S6E). Collectively, the suppression of SMAD9 attenuates MYCN-amplified NB growth both in vitro and in vivo.

### The SMAD9 binding pattern is associated with MYCN

**Fig. 5:**
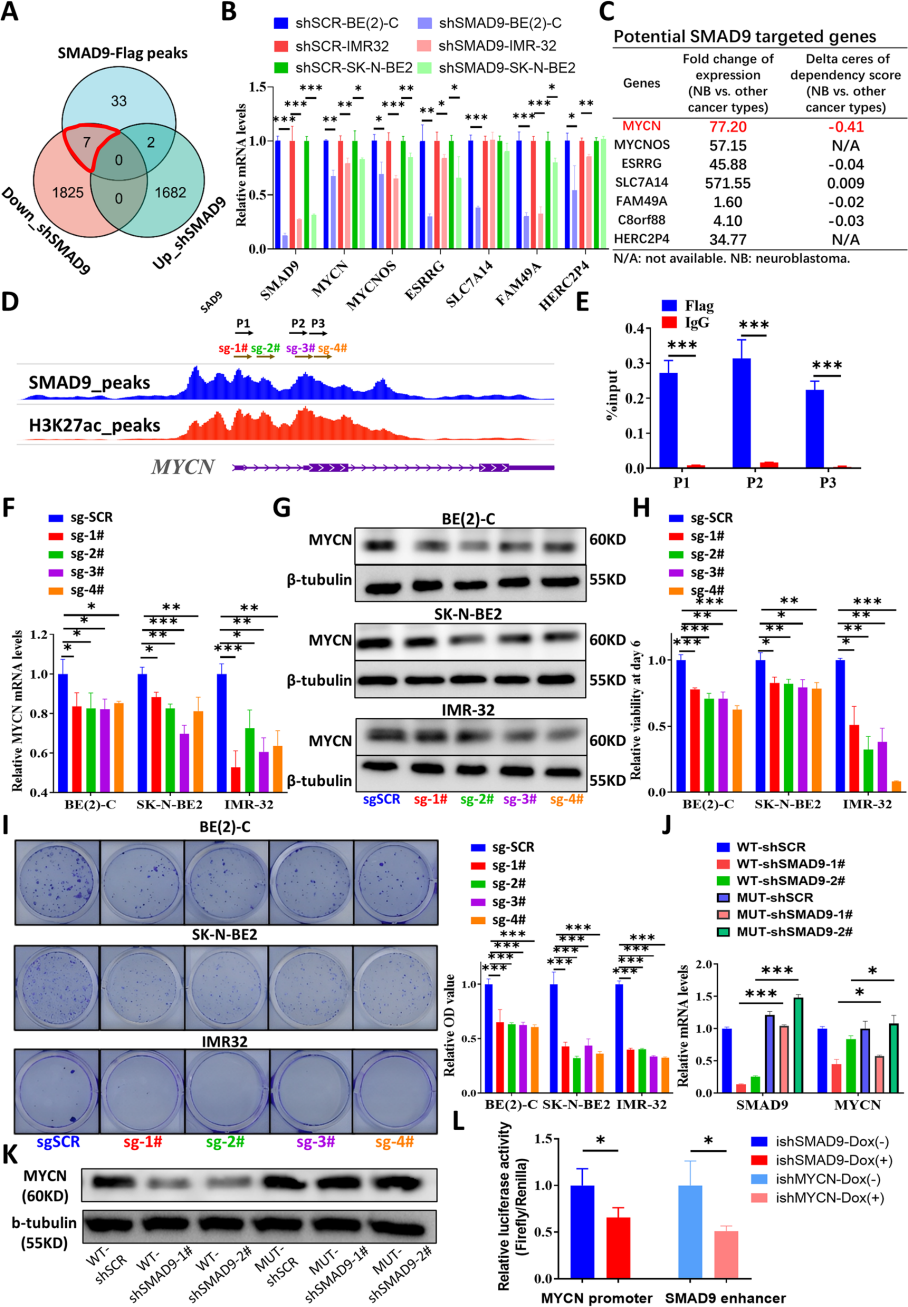
The SMAD9 binding pattern is associated with MYCN. **A** Venn diagram showing the 7 overlapping downregulated genes between the ChIP-seq and RNA-seq results in BE(2)-C cells. **B** Q-RT-PCR analyses of the overlapping gene expression after SMAD9 knockdown in MYCN-amp NB cells. **C** Table showing the expression and dependency profile of 7 overlapping genes based on the DepMap portal. **D, E** Gene track showing high binding signals for SMAD9 and H3K27ac in the MYCN promoter region detected using ChIP-seq (**D**). P1, P2 and P3 indicate the primer locations for ChIP-QPCR (**D**), and the results are shown (**E**). Sg-1#, 2#, 3# and 4# indicate the sgRNA primers based on the locations for subsequent CRISPRi experiments (**D**). **(F, G)** Q-RT-PCR (**F**) and immunoblotting (**G**) analyses of MYCN expression after disrupting the binding of SMAD9 to MYCN with the CRISPRi system in MYCN-amp dCas9 STCs. **H, I** Cell viability on day 6 (**H**), representative images of the colony formation (**I**, left panel) and their quantification (**I**, right panel) for MYCN-amp dCas9 STCs with disrupted binding of SMAD9 to MYCN. **J, K** SMAD9 rescue experiments showing recovered SMAD9 and MYCN expression detected using Q-RT-PCR (**J**), and recovered MYCN expression detected using immunoblotting (**K**). **L** Relative luciferase activity of the luciferase reporter gene containing the MYCN promoter or SMAD9 enhancer in BE(2)-C cells with SMAD9 knockdown or MYCN knockdown. ChIP: chromatin immunoprecipitation; ChIP-seq: ChIP sequencing; dCas9: dead Cas9; ish: inducible shRNA; MYCN-amp: MYCN amplification; sgSCR: sgRNA scrambled control; shSCR: shRNA scrambled control; STCs: stably transfected cells. *P < 0.05, **P < 0.01, and *** *P* < 0.001

Next, we investigated the transcriptional role of SMAD9 (Fig. S7A). Due to the lack of commercially reliable anti-human SMAD9 antibodies, we constructed SMAD9 overexpression constructs with the 3xFlag tag plasmid and established stably transfected MYCN-amplified BE(2)-C cells (Figs. S7B and S7C). We conducted ChIP-seq for BE(2)-C-SMAD9-Flag STCs, combined the results of RNA-seq for BE(2)-C-shSMAD9 cells and found 7 overlapping genes (Fig. 5A). Q-RT-PCR analyses validated that these genes were potential downstream genes (Fig. 5B). Based on the expression and dependency data from the DepMap portal, we found that MYCN was the only gene that exhibited a high and specific dependency on NB (Fig. 5C).

Specifically, we visualized strong binding for SMAD9 and H3K27ac in the MYCN promoter region (Fig. 5D). ChIP-QPCR experiments validated the aforementioned finding (Fig. 5E). CRISPRi experiments showed that blocking SMAD9 binding sites suppressed MYCN mRNA (Fig. 5F) and protein expression (Fig. 5G) in three types of MYCN-amplified dCas9 STCs. In parallel, the disruption of SMAD9 binding sites for MYCN diminished cell proliferation (Fig. 5H) and inhibited colony formation in MYCN-amplified dCas9 STCs (Figs. 5I and S7D). Furthermore, the recovery of SMAD9 expression restored MYCN mRNA (Fig. 5J) and protein expression (Fig. 5K). We further validated the transcriptional feedback loop between SMAD9 and MYCN by constructing a luciferase reporter containing the MYCN promoter or SMAD9 enhancer. The dual-luciferase reporter analysis showed that SMAD9 or MYCN knockdown reduced the activity of MYCN promoter or SMAD9 enhancer (Fig. 5L).

Interestingly, we also detected a strong binding signal for SMAD9 and H3K27ac in the MYCNOS promoter region (Fig. S7D). MYCNOS expression was higher in high-risk NB tissues than in nonhigh-risk NB tissues, and higher in MYCN-amp NB tissues than in nonMYCN-amp NB tissues (Figs. S7E and S7F). Disrupted binding sites of SMAD9 to MYCN also suppressed MYCNOS transcription (Fig. S7G) and the recovery of SMAD9 expression restored MYCNOS mRNA levels (Fig. S7H).

**Fig. 6:**
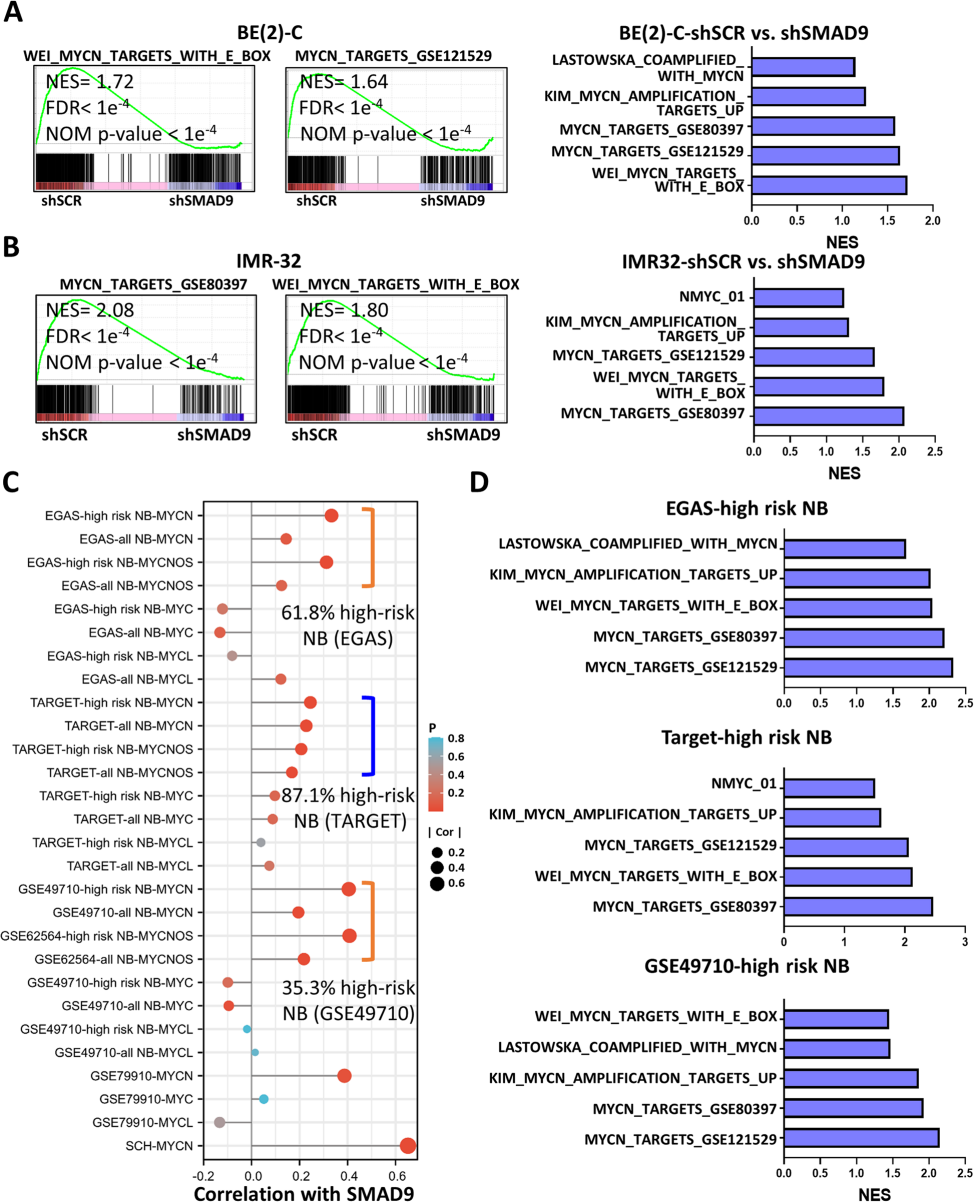
MYCN-associated GSEA signatures in SMAD9-high NB cells and tumors. **A, B** Representative GSEA of MYCN-associated gene sets in the SMAD9-high and SMAD9-low groups among BE(2)-C (**A**) and IMR-32 cells (**B**) with FDR and NOM p values. **C** Lollipop chart showing the correlation value between SMAD9 and MYCN (or MYCNOS or MYC family members) in different datasets and the SCH cohort. **D** Representative GSEA of MYCN-associated gene sets in the SMAD9-high and SMAD9-low groups in different high-risk NB cohorts. EGAS: dataset EGAS00001001308; GSEA: gene set enrichment analysis; shSCR: shRNA scrambled control; SCH: Shanghai Children Hospital; TARGET: therapeutically applicable research to generate effective treatments

Similar to the results from ChIP-seq, the GESA of RNA-seq data from BE(2)-C and IMR-32 cells showed that high SMAD9 expression had a high normalized enrichment score in the terms of “WEI_MYCN_TARGETS_WITH_E_BOX”, “KIM_MYCN_AMPLIFICATION_UP”, “MYCN_TARGETS_GSE121529”, “MYCN_TARGETS_GSE80397” and other MYCN-associated signatures (Fig. 6A and B). In addition to NB cells, we observed a positive correlation between SMAD9 and MYCN (or MYCNOS) expression in high-risk NB tumors and a much lower correlation in the NB cohorts that included fewer high-risk patients (Fig. 6C), consistent with previous results (Figs. 1D and S2E-G). GSEA of NB tissues showed that high expression of SMAD9 had a high enrichment score in these MYCN-associated signatures (Fig. 6D).

Taken together, our results indicate that SMAD9 acts as a TF and is capable of binding MYCN to promote MYCN-amplified NB cell growth and that SMAD9-MYCN forms a positive transcriptional feedback loop.

### Malignant cell cycle changes occur in response to SMAD9 knockdown

**Fig. 7:**
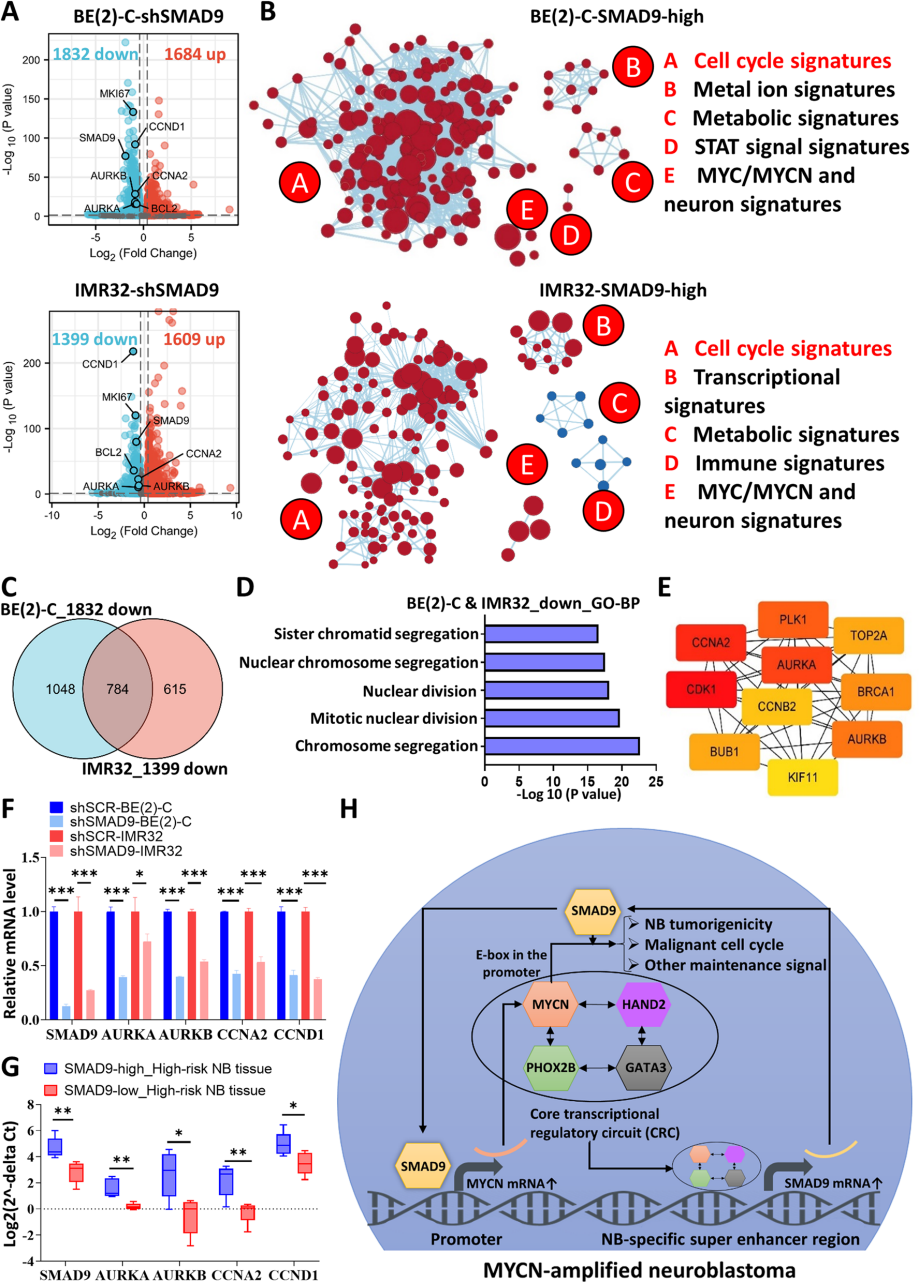
Transcriptome changes in response to SMAD9 knockdown. **A** Volcano plots showing the DEGs in BE(2)-C and IMR-32 cells after SMAD9 knockdown. The cutoff setting was |log_2_FC| > 0.4, *P* < 0.05. **B** Functional enrichment map depicting the functional groups of the GSEA hits for the SMAD9-high effects on BE(2)-C and IMR-32 cells based on shSMAD9 RNA-seq results. **C** Venn diagram showing the 784 overlapping downregulated genes between BE(2)-C and IMR-32 cells. **D** Top 5 gene sets functionally enriched in GO-BP terms. **E** The “Degree” approach using Cytoscape software (CytoHubba plugin) to show the top 10 hub genes in the overlapping 784 downregulated genes. **F, G** Q-RT-PCR validation of some hub genes in NB cells (**F**) and NB tissue samples (**G**) from SCH that were classified by the median expression of SMAD9. **H** Summary diagram describing the positive feedback loop between SMAD9 and MYCN and the MYCN-induced cancer cell cycle in MYCN-amplified NB cells. DEGs: differentially expressed genes; GO-BP: Gene Ontology, biological process; GSEA: gene set enrichment analysis; NB: neuroblastoma; Q-RT-PCR: quantitative reverse transcription polymerase chain reaction; SCH: Shanghai Children’s Hospital. *P < 0.05, **P < 0.01, and *** *P* < 0.001

Finally, we performed unbiased transcriptomic analyses of shSCR and shSMAD9 samples among BE(2)-C and IMR-32 cells, with > 25% decreases in MKI67, BCL2 and CCND1 levels (Fig. 7A). GSEA showed that high SMAD9 expression was mainly enriched in terms related to the malignant cell cycle both in BE(2)-C and IMR-32 cells (Figs. 7B and S8A-B). Therefore, we combined the RNA-seq results from the two cell lines and subsequently investigated the 784 overlapping downregulated genes (Fig. 7C). GO/KEGG analyses revealed that the 784 genes were mainly enriched in cancer cell cycle terms such as “chromosome segregation” and “mitotic nuclear division” in GO-biological processes (GO-BP) analysis (Fig. 7D). Similarly, other signatures, such as “condensed chromosome” in GO cellular component (GO-CC) terms, “microtubule binding” in GO molecular function (GO-MF) terms and “cell cycle” in KEGG pathways, revealed the significance of phenotypic malignancy (Fig. S8C). Regarding upregulated genes in shSMAD9 cells, we identified 570 common upregulated genes, and GO/KEGG analyses suggested the enrichment of mature nervous signatures such as “modulation of chemical synaptic transmission” in GO-BP terms and “neuron to neuron synapse” in GO-CC terms (Fig. S8D).

The common downregulated genes were subjected to PPI analysis to further identify the downregulated hub genes, and we found that AURKA-linked cell cycle and mitotic nuclear division genes such as AURKA, AURKB and CCNA2 might play a pivotal role in SMAD9-MYCN-mediated malignancy (Fig. 7E), which was validated by performing Q-RT-PCR analysis of NB cells (Fig. 7F) and tissues from SCH (Fig. 7G). We briefly validated previous findings concerning the MYCN and cell cycle pathways [30–32] by analyzing two datasets from MYCN knockdown cells and found that most of SMAD9-high hub genes had comparable edges in MYCN-high NB cells (Figs. S9A and S9B). The validated cell cycle genes were downregulated in MYCN-low NB cells and tumors (Fig. S9C). Notably, the ChIP-seq data for MYCN suggested the significance of E-box motifs (CACGTG or CANNTG) in MYCN-amp NB cells (Figs. S9D and S9E). We identified abundant E-box sequences in the promoters of the hub cell cycle genes in different types of MYCN-amp NB cells (Figs. S9F-S9G).

The combined analysis of SMAD9 binding patterns and transcriptomic changes in SMAD9 knockdown cells indicated that SMAD9 might contribute to MYCN-mediated autonomous tumorigenicity and the cancer cell cycle in MYCN-amplified NB. As described previously, SMAD9 is a NB-specific SEs-targeted gene mediated by MYCN and other TFs in CRC and dual luciferase assays showed that SMAD9 or MYCN knockdown reduced the activity of MYCN promoter or SMAD9 enhancer. Collectively, the positive feedback loop between SMAD9 and MYCN reinforces autonomous nervous system tumorigenicity and the tumor cancer cycle in MYCN-amplified NBs (Fig. 7H).

## Discussion

Despite the use of multimodal therapeutic strategies, COG-defined high-risk NB has a low 5-year OS rate [33]. NB is driven by the interplay of regulatory TFs and dynamic alterations in chromatin structure [34]. Therefore, in this study, we performed integrative analyses of public datasets and identified SMAD9 as a potential prognostic risk factor in the high-risk NB patients. In addition, SMAD9 was shown to be a SEs-targeted gene in NB, and we revealed SMAD9 as a regulator of MYCN-amplified NB cell proliferation and tumorigenicity. Mechanistic studies showed that SMAD9 bound to the MYCN promoter, partially regulated MYCN expression and further mediated the cancer cell cycle. Taken together, MYCN, GATA3, PHOX2B and HAND2 reciprocally triggered SMAD9 transcription by binding its enhancers, thus forming a positive feedback loop between SMAD9 and MYCN.

TGF-beta/SMAD signaling plays an important role in tumor development, relapse, drug resistance and metastasis [35–40]. The 8 types of SMAD proteins constitute 3 functional groups: receptor-regulated SMADs (R-SMADs; which include SMAD2/3 and SMAD1/5/9), co-mediator SMAD (Co-SMAD; which include SMAD4) and inhibitory SMADs (I-SMADs; which includes SMAD6, 7). A Co-SMAD forms a heteromeric complex with an R-SMAD and translocates into the nucleus to regulate gene transcription [35]. Ekaterini et al. [41] documented that activin A activated the ALK4/SMAD2/3 pathway in endothelial cells and attenuated NB xenografts, indicating that SMAD2/3 function as tumor suppressors in NB. Nevertheless, Hung et al. [42] showed that SMAD2 potentially promoted NB. Several reports have examined the roles of SMAD1/5 in NB [43, 44]. However, the role and mechanism of SMAD9 in NB remain to be elucidated.

MYCN has been implicated in NB development [30], drug resistance [45] and metastasis [46]. Recent studies have aimed to identify targets that indirectly suppress MYCN expression [21, 34, 47–49]. To our knowledge, this study is the first to identify a direct link between SMAD9 and MYCN. First, we found that SMAD9 had a specific expression and dependency profile in NB and showed a positive correlation with MYCN expression. Next, we showed that MYCN coordinated with other TFs in CRC to target SMAD9 enhancers and transcriptionally activate SMAD9 expression. In addition, SEs-targeted SMAD9 promoted the growth of MYCN-amplified NB cells both in vitro and in vivo. Furthermore, a combination of ChIP-seq, RNA-seq, and CRISPRi analyses indicated that SMAD9 targeted the MYCN promoter and transcriptionally activated MYCN expression. Along with the dual-luciferase reporter assays, our results highlight the significance of the positive feedback loop between SMAD9 and MYCN. Some of the existing positive feedback loops between other tumor-dependent genes and MYCN have been reported [21, 34], and our study has provided additional evidence for MYCN networks.

It has been documented that MYCNOS functions as an antisense RNA and results in a reduction of upstream MYCN promoter usage and thus upregulates MYCN expression [50]. In addition, CCCTC-binding factor (CTCF) cooperates with noncoding RNA MYCNOS to promote neuroblastoma progression through facilitating MYCN expression [51]. We identified a strong binding for SMAD9 and H3K27ac in the MYCNOS promoter region and the recovery of SMAD9 increased MYCNOS expression, suggesting that MYCNOS might be critical in the SMAD9-MYCN positive feedback loop.

MYCN induces quiescent cells to reenter the cell cycle and shortens the cell cycle time, in particular shortening G1 phase [30, 31, 52–54]. SMAD9 knockdown attenuated the transcriptomic phenotypes of MYCN-associated autonomous nervous system development and the cancer cell cycle. We identified overlapping cell cycle hub genes, such as AURKA and CCNA2, in both MYCN and SMAD9 knockdown NB cells. And we validated the E-box sequences in the promoter of the hub cell cycle genes. Taken together, these results suggest that SMAD9 potentially mediates the MYCN-relevant cell cycle in a subset of high-risk NB cells.

Some limitations of our study should be noted. We only used BE(2)-C cells for in vivo experiments because other NB cells exhibited less tumorigenic potential and high heterogeneity in nude mice. Although we identified a possible positive feedback loop between SMAD9 and MYCN, our current study did not precisely delineate the crosstalk between the SMAD9 pathway and MYCN expression**.** Notably, due to the lack of a reliable anti-human SMAD9 antibody, we constructed a SMAD9-Flag-tagged vector in MYCN-amplified cells to perform ChIP-seq analysis according to previous reports [55, 56]. Although our results revealed a low number of SMAD9-Flag binding peaks, we did not obtain evidence for false positive or negative binding events in Flag ChIP-seq experiments [57].

In summary, high SMAD9 expression in NB was specifically regulated by multiple TFs at the SEs region in CRC. In addition, SMAD9 knockdown exerted an antitumor effect on MYCN-amplified NB by disrupting the positive feedback loop between SMAD9 and MYCN. These findings will help develop novel diagnostic and therapeutic strategies for high-risk NB.

## Conclusion

SMAD9 forms a positive transcriptional feedback loop with MYCN and represents a unique tumor dependency for MYCN-amplified neuroblastoma.

## Supplementary Information

**Additional file 1: Fig. S1.** Workflow of the study. **Fig. S2.** Integrative screen revealing NB-specific expression and dependency on SMAD9. **Fig. S3.** SMAD9 is an indicator of a poor prognosis in a subset of high-risk patients with NBs. **Fig. S4.** SMAD9 is a SEs-targeted gene in NB and knockdown of the NB-specific gene in CRC inhibits SMAD9 expression. **Fig. S5.** SMAD9 profile in our NB samples and PDC growth suppression upon SMAD9 knockdown. **Fig. S6.** Doxycycline induces SMAD9 knockdown in vitro and in vivo. **Fig. S7.** The workflow of ChIP-seq and MYCNOS evaluations in high-grade NB tissues and cells. **Fig. S8.** Transcriptome changes in response to SMAD9 knockdown. **Fig. S9.** Transcriptome changes in response to MYCN knockdown and binding patterns of E-box sequences.

**Additional file 2.**

**Additional file 3.**

**Additional file 4.**

**Additional file 5.**

**Additional file 6.**

**Additional file 7.**

## Data Availability

The data in this study are available from the corresponding author on reasonable request. And the datasets are available in the following databases. Microarray data: GSE12460 (https://www.ncbi.nlm.nih.gov/geo/query/acc.cgi?acc=GSE12460), GSE13136(https://www.ncbi.nlm.nih.gov/geo/query/acc.cgi?acc=GSE13136), GSE16476(https://www.ncbi.nlm.nih.gov/geo/query/acc.cgi?acc=GSE16476), GSE49710(https://www.ncbi.nlm.nih.gov/geo/query/acc.cgi?acc=GSE49710), GSE79910(https://www.ncbi.nlm.nih.gov/geo/query/acc.cgi?acc=GSE79910), GSE28019(https://www.ncbi.nlm.nih.gov/geo/query/acc.cgi?acc=GSE28019), GSE121529(https://www.ncbi.nlm.nih.gov/geo/query/acc.cgi?acc=GSE121529), GSE90121(https://www.ncbi.nlm.nih.gov/geo/query/acc.cgi?acc=GSE90121), GSE3526(https://www.ncbi.nlm.nih.gov/geo/query/acc.cgi?acc=GSE3526), GSE7307(https://www.ncbi.nlm.nih.gov/geo/query/acc.cgi?acc=GSE7307), GSE8514(https://www.ncbi.nlm.nih.gov/geo/query/acc.cgi?acc=GSE8514), GSE14340(https://www.ncbi.nlm.nih.gov/geo/query/acc.cgi?acc=GSE14340) and TARGET (https://hgserver1.amc.nl/r2/tables/ps_avgpres_targetnrbl249_huex10t_box1659092437-datagrabber-.txt). RNA-seq data: GSE80397 (https://www.ncbi.nlm.nih.gov/geo/query/acc.cgi?acc=GSE80397), GSE124451(https://www.ncbi.nlm.nih.gov/geo/query/acc.cgi?acc=GSE124451) and EGAS00001001308(https://hgserver1.amc.nl/r2/tables/ps_avgpres_2010fwr144_gencode19_box1659091546-datagrabber-.txt). ChIP-seq data: GSE94822 (https://www.ncbi.nlm.nih.gov/geo/query/acc.cgi?acc=GSE94822). Depmap database (https://depmap.org/portal/; version 22Q1). Super-enhancer database (SEdb; http://www.licpathway.net/sedb/).

## Abbreviations

AG: Adrenal gland
BP: Biological process
CC: Cell component
CDS: Coding sequence
ChIP: Chromatin immunoprecipitation
COG: Children oncology group
CRC: Core (transcriptional) regulatory circuit
CRISPRi: CRISPR interference
CTCF: CCCTC binding factor
CTG: Cell titer Glo
CVS: Crystal violet staining
dCas9: Dead Cas9
Dox: Doxycycline
EGAS: Dataset EGAS00001001308
EV: Empty vector
EVS: External Validation set
FC: Fold change
FDR: False discovery rate
GO: Gene ontology
GSEA: Gene set enrichment analysis
HE: Hematoxylin and eosin
IHC: Immunohistochemistry
ish: Inducible shRNA
KEGG: Kyoto encyclopedia of genes and genomes
MF: Molecular function
MYCN-amp: MYCN amplification
MOI: Multiplicity of infection
N/A: Not available
NB: Neuroblastoma
NES: Normalized enrichment score
nonMYCN-amp: Non-MYCN amplification
NOM: Normalized
NTC: Nontargeted control
PDC: Patient derived cells
PDX: Patient derived xenograft
PPI: Protein to protein interaction
Q-RT-PCR: Quantitative reversed transcription polymerase chain reaction
SCH: Shanghai children’s hospital
SE: Super-enhancers
sgRNA: Small guide RNA
sgSCR: sgRNA scrambled control
shRNA: Short hairpin RNA
shSCR: shRNA scrambled control
STCs: Stable transfected cells
TF: Transcription factor
WT: Wild type
