# Supplementary material for: SMAD9-MYCN positive feedback loop represents a unique dependency for MYCN-amplified neuroblastoma
Source: J Exp Clin Cancer Res. 2022 Dec 20;41:352. doi: 10.1186/s13046-022-02563-3 (PMC9764568; doi:10.1186/s13046-022-02563-3)
Supplement: Supplementary file 1 — Additional file 1: Fig. S1. Workflow of the study. Fig. S2. Integrative screen revealing NB-specific expression and dependency on SMAD9. Fig. S3. SMAD9 is an indicator of a poor prognosis in a subset of high-risk patients with NBs. Fig. S4. SMAD9 is a SEs-targeted gene in NB and knockdown of the NB-specific gene in CRC inhibits SMAD9 expression. Fig. S5. SMAD9 profile in our NB samples and PDC growth suppression upon SMAD9 knockdown. Fig. S6. Doxycycline induces SMAD9 knockdown in vitro and in vivo. Fig. S7. The workflow of ChIP-seq and MYCNOS evaluations in high-grade NB tissues and cells. Fig. S8. Transcriptome changes in response to SMAD9 knockdown. Fig. S9. Transcriptome changes in response to MYCN knockdown and binding patterns of E-box sequences. [file 13046_2022_2563_MOESM1_ESM.docx]

**Supplementary figures**


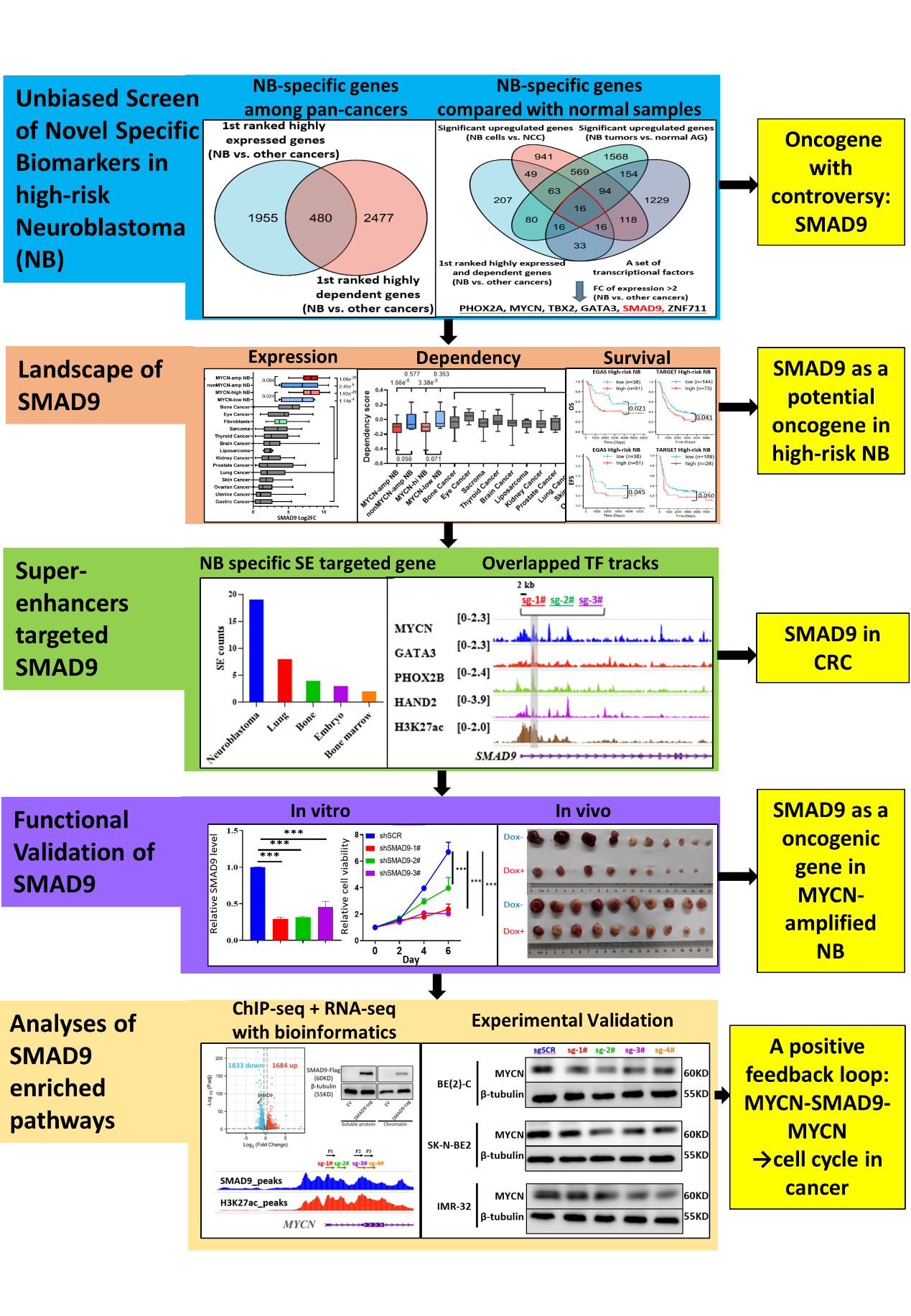


**Figure S1. Workflow of the study.**

**
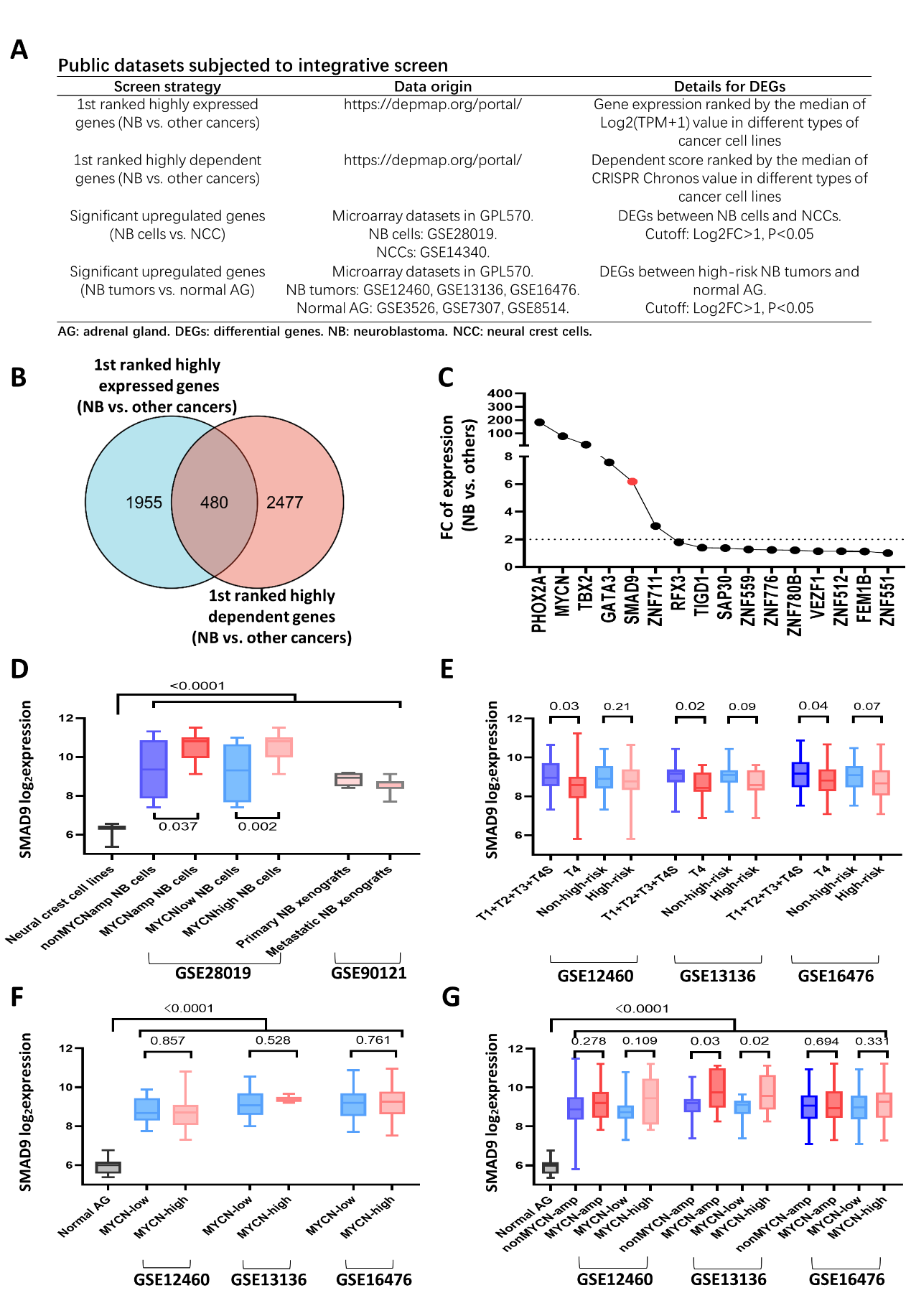
**

**Figure S2. Integrative screen revealing NB-specific expression and dependency on SMAD9.**

***(A)*** *Table showing the details of the integrative screen.* ***(B)*** *Venn diagram showing the 480 overlapping genes based on the 1^st^ ranked highly expressed and dependent genes in the Depmap portal.* ***(C)*** *Ranks of 16 NB-specific transcripts based on the FC in expression.* ***(D)*** *SMAD9 expression profile in normal and NB cells in the GPL570 microarray platform.* ***(E)*** *SMAD9 expression profile in NB tumors that were classified by INSS and COG risk groups.* ***(F, G)*** *SMAD9 expression profile in nonhigh-risk NB (F) and all NB (G) tumors in the GPL570 microarray platform. The MYCN-high and MYCN-low groups were classified based on the median expression of MYCN.*

*AG: adrenal gland; COG: Children’s Oncology Group; FC: fold change; INSS: International Neuroblastoma Staging System; MYCN-amp: MYCN amplification; NB: neuroblastoma; NCC: neural crest cells.*

**
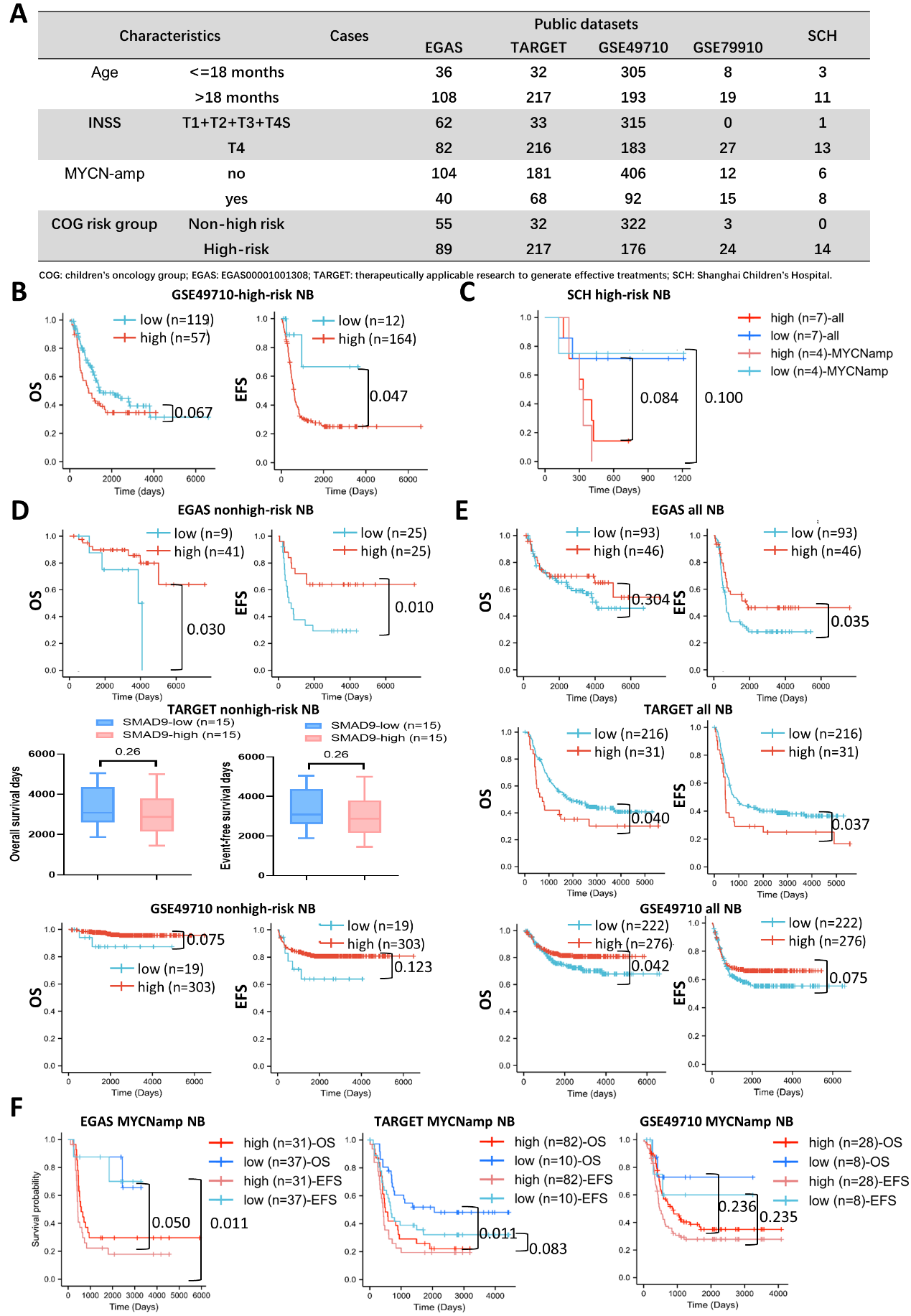
**

**Figure S3. SMAD9 is an indicator of a poor prognosis in a subset of high-risk patients with NBs.**

***(A)*** *Table showing the characteristics of NB datasets and NB tumors from SCH.* ***(B, C)*** *Kaplan-Meier curves of the SMAD9-high group and SMAD9-low group in high-risk NB patients from GSE49710 (B) and SCH (C).* ***(D, E)*** *Kaplan-Meier curves of the SMAD9-high group and SMAD9-low group in nonhigh-risk NB patients (D) and all NB patients (E) from EGAS, TARGET and GSE49710. As the nonhigh-risk patients in the TARGET cohort were alive and free of events, the bar plot showed survival (days) in the SMAD9-high and SMAD9-low groups (middle panel, D).* ***(F)*** *Kaplan-Meier curves of the SMAD9-high group and SMAD9-low group of MYCN-amp NB patients from EGAS, TARGET and GSE49710.*

*COG: Children’s Oncology Group; EFS: event-free survival; EGAS: dataset EGAS00001001308; FC: fold change; INSS: International Neuroblastoma Staging System; MYCN-amp: MYCN amplification; NB: neuroblastoma; NCC: neural crest cells;* *OS: overall survival; TARGET: therapeutically applicable research to generate effective treatments. SCH: Shanghai Children’s Hospital*

*
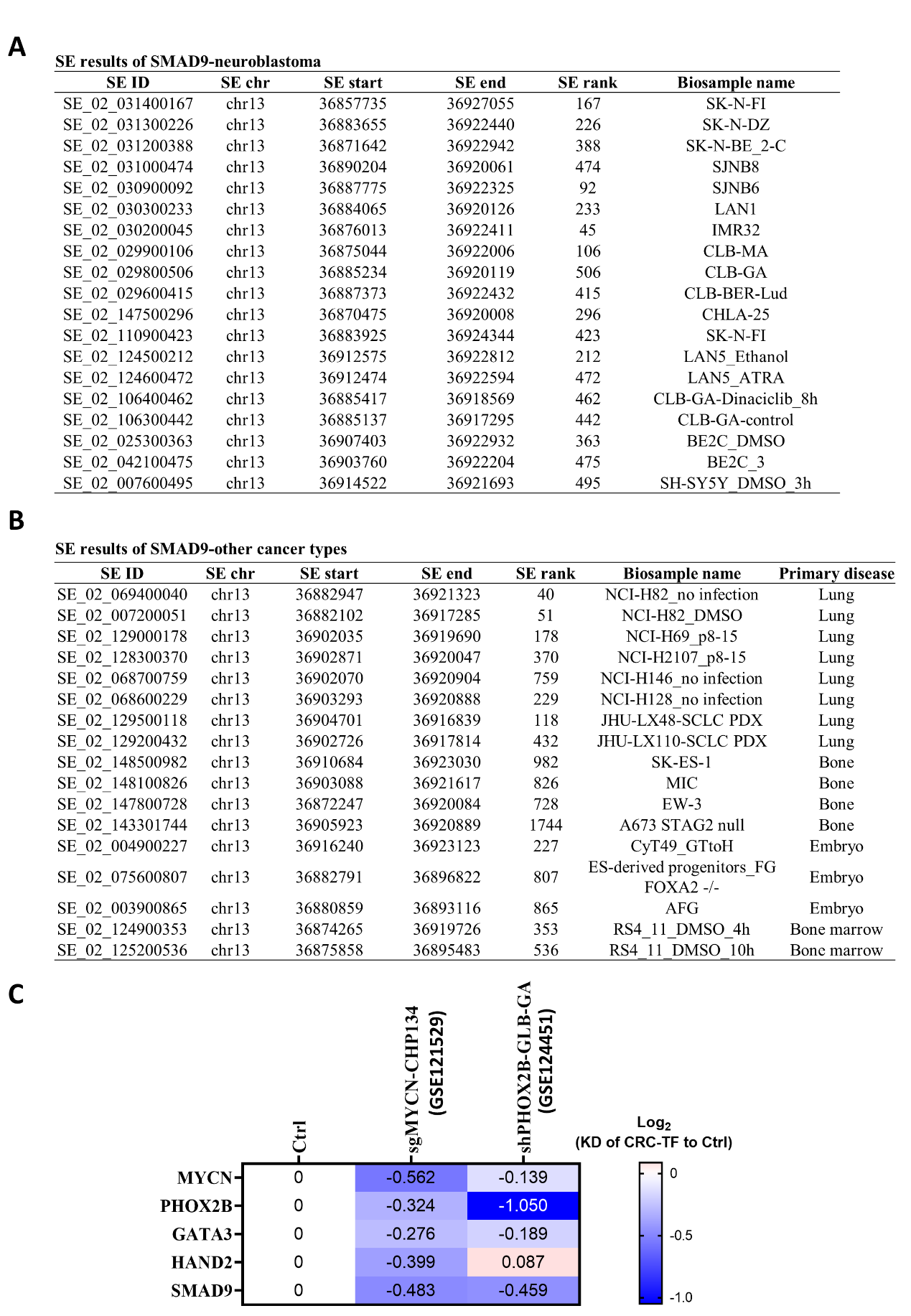
*

**Figure S4. SMAD9 is a SEs-targeted gene in NB and knockdown of the NB-specific gene in CRC inhibits SMAD9 expression.**

***(A, B)*** *The tables showing SEs results for SMAD9 in NB (A) and other cancer types (B).* ***(C)*** *Heatmap showing bioinformatic analyses of changes in MYCN, GATA3, PHOX2B, HAND2 and SMAD9 expression after MYCN knockdown in CHP134 cells (GSE121529) and PHOX2B in GLB-GA cells (GSE124451).*

*NB: neuroblastoma;* *SE: super-enhancers.*

**
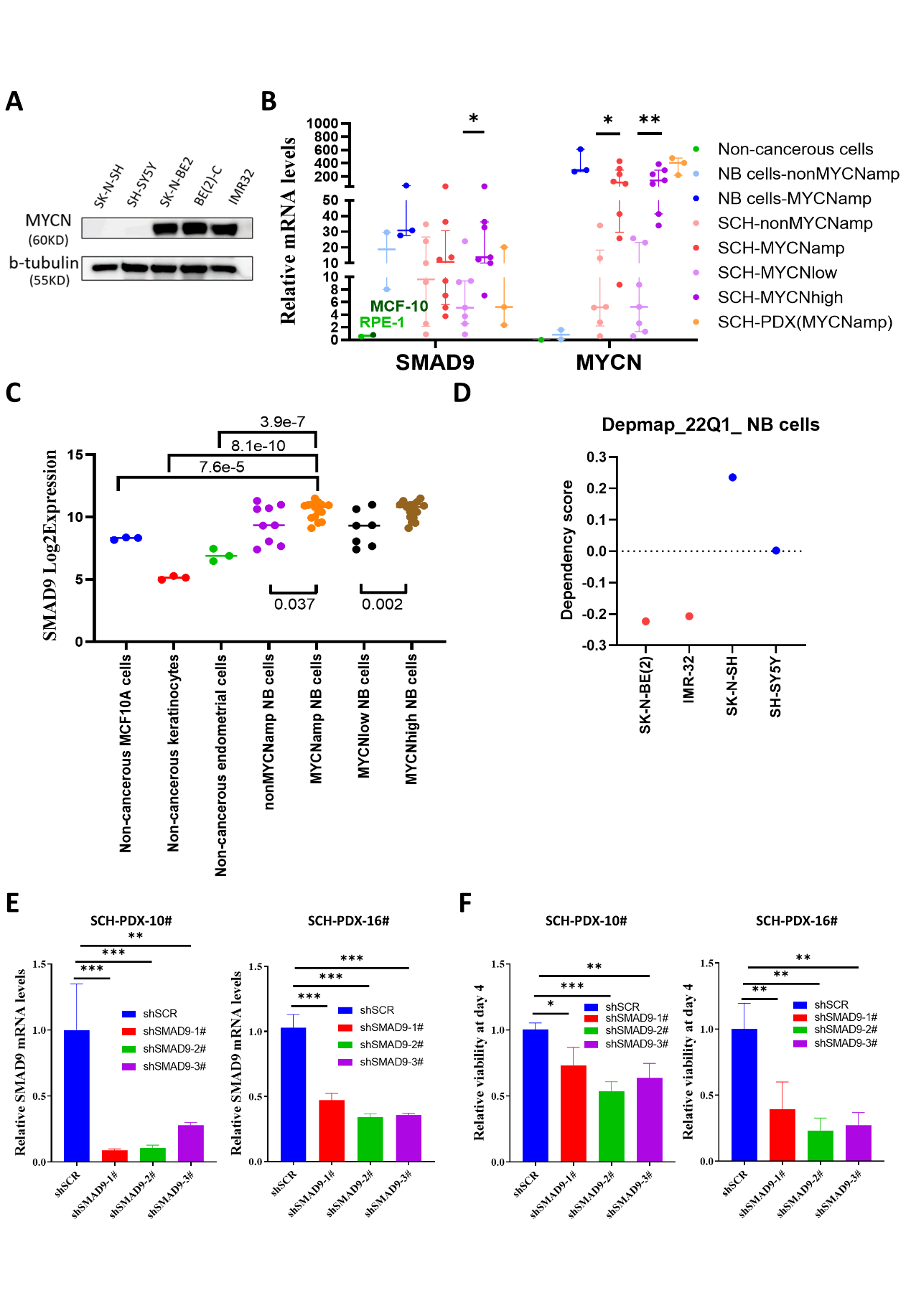
**

**Figure S5. SMAD9 profile in our NB samples and PDC growth suppression upon SMAD9 knockdown.**

***(A)*** *MYCN expression profile in NB cell lines.* ***(B)*** *MYCN and SMAD9 mRNA levels in NB cell lines (MYCN-amp cells: BE2-C, SK-N-BE2 and IMR-32; non-MYCN-amp cells: SK-N-SH and SH-SY5Y), noncancerous cell lines (neuro-ectodermal RPE-1 cells and epithelium-derived MCF-10 cells), SCH NB and SCH NB PDX tissues.* *The MYCN-high and MYCN-low groups were classified based on the median expression of MYCN.* ***(C)*** *SMAD9 expression in microarray datasets among different types of cells.* ***(D)*** *The dependency score of NB cell lines in the DepMap database.* ***(E, F)*** *Knockdown (E) and growth curve (f) of PDCs extracted from high-risk NB patients (10# and 16#) in SCH.*

*MYCN-amp: MYCN amplification; NB: neuroblastoma; PDCs: patient derived cells; PDX: patient derived xenograft; shSCR: shRNA scrambled control. *P<0.05, ** P<0.01, and *** P<0.001.*

*
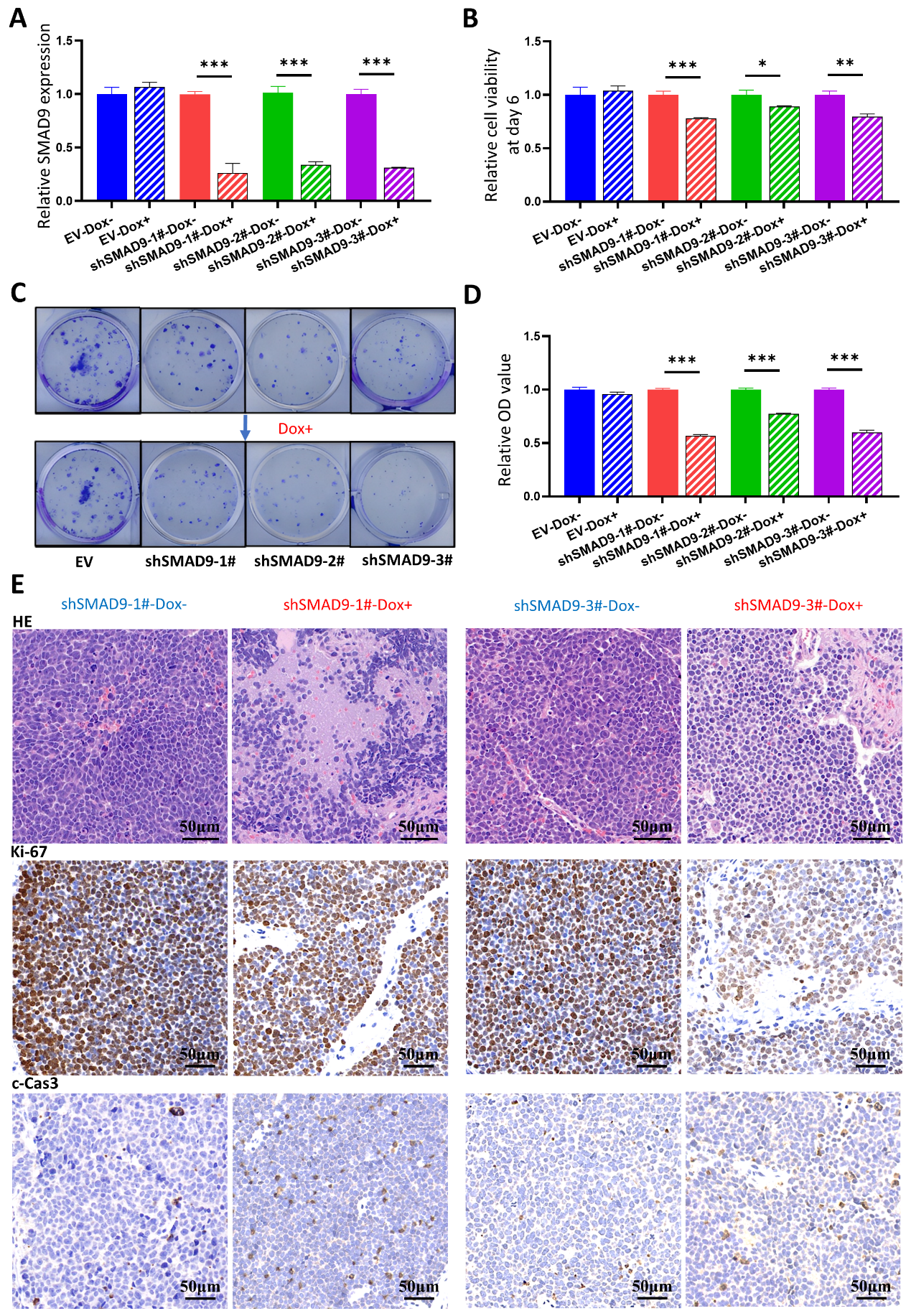
*

**Figure S6. Doxycycline induces SMAD9 knockdown in vitro and in vivo.**

***(A, B)*** *SMAD9 knockdown (A) in BE(2)-C cells stably transfected with EV and Dox(Tet)-on-shSMAD9-1#, 2# and 3# constructs and cell viability on day 6 in vitro (B).* ***(C, D)*** *Representative images of colonies formed for stably transfected BE(2)-C cells (C) and crystal violet staining (D).* ***(E)*** *Representative images (20x magnification) of HE and IHC (Ki-67 for proliferation and c-Cas3 for apoptosis) staining in tumor tissues from shSMAD9 Dox-off and Dox-on mice.*

*c-Cas3: cleaved caspase 3; Dox: doxycycline; EV: empty vector; HE: hematoxylin and eosin; IHC: immunohistochemistry. * P<0.05, ** P<0.01, and *** P<0.001.*

**
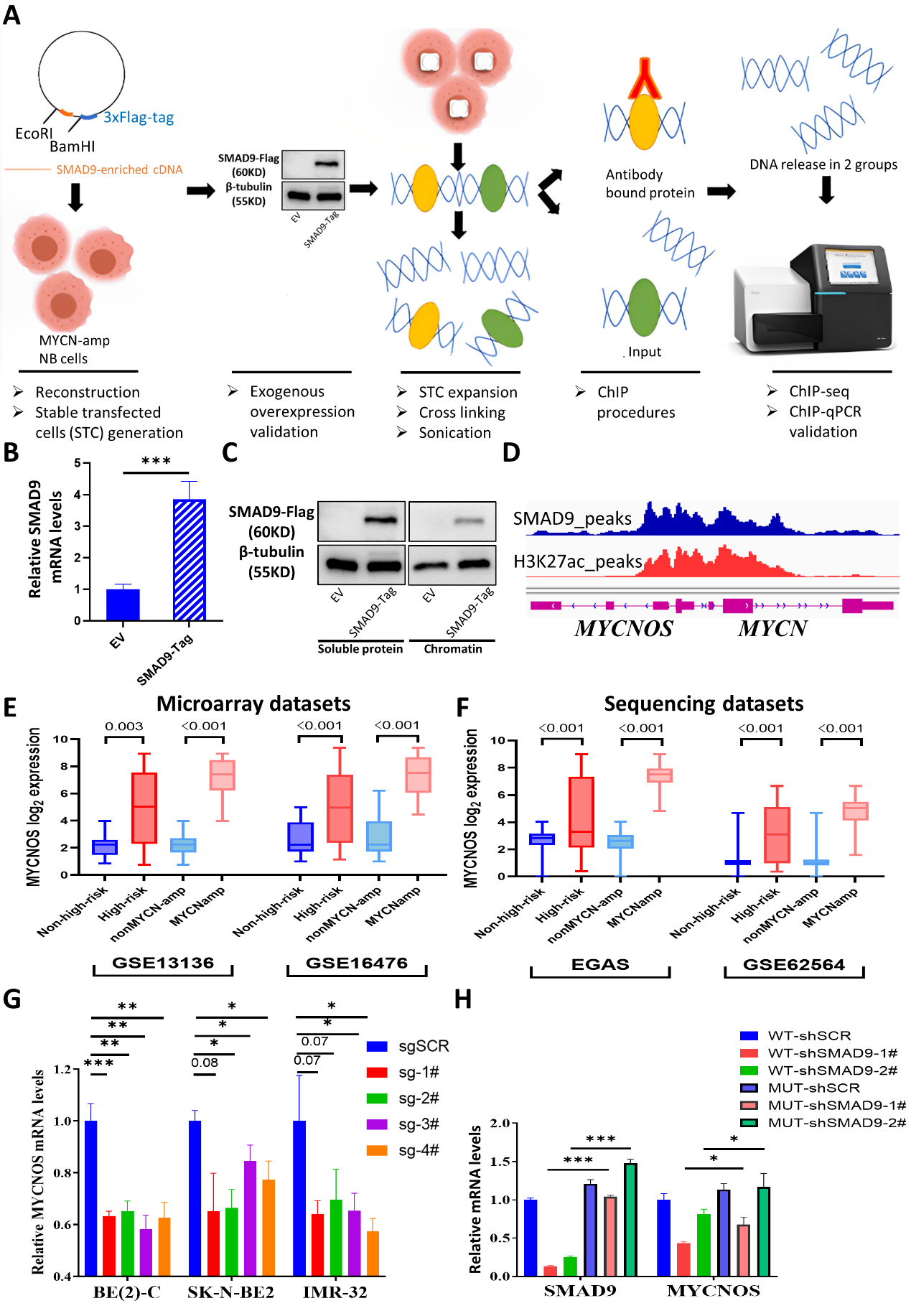
**

**Figure S7. The workflow of ChIP-seq and MYCNOS evaluations in high-grade NB tissues and cells.**

***(A)*** *ChIP-seq workflow in exogenously overexpressed SMAD9-Flag-tagged STCs.* ***(B, C)*** *SMAD9 expression detected using Q-RT-PCR, and (C) Flag-tagged proteins detected using Western blot analysis in MYCN-amplified NB STCs.* ***(D)*** *Gene track showing high binding signals for SMAD9 and H3K27ac in the MYCNOS promoter region detected using ChIP-seq.* ***(E, F)*** *MYCNOS expression in nonhigh-risk and high-risk NB tissues, as well as nonMYCN-amp and MYCN-amp NB tissues in microarray (E) and sequencing (F) datasets.* ***(G)*** *Q-RT-PCR analyses of MYCNOS expression after disrupting the binding of SMAD9 to MYCN with the CRISPRi system in MYCN-amp dCas9 STCs.* ***(H)*** *SMAD9 rescue experiments showing recovered SMAD9 and MYCNOS expression, as measured using Q-RT-PCR.*

*EV: empty vector; MYCM-amp: MYCN amplification; NB: neuroblastoma; sgSCR: sgRNA scrambled control; STC: stably transfected cells; *P<0.05, **P<0.01, and *** P<0.001.*

***
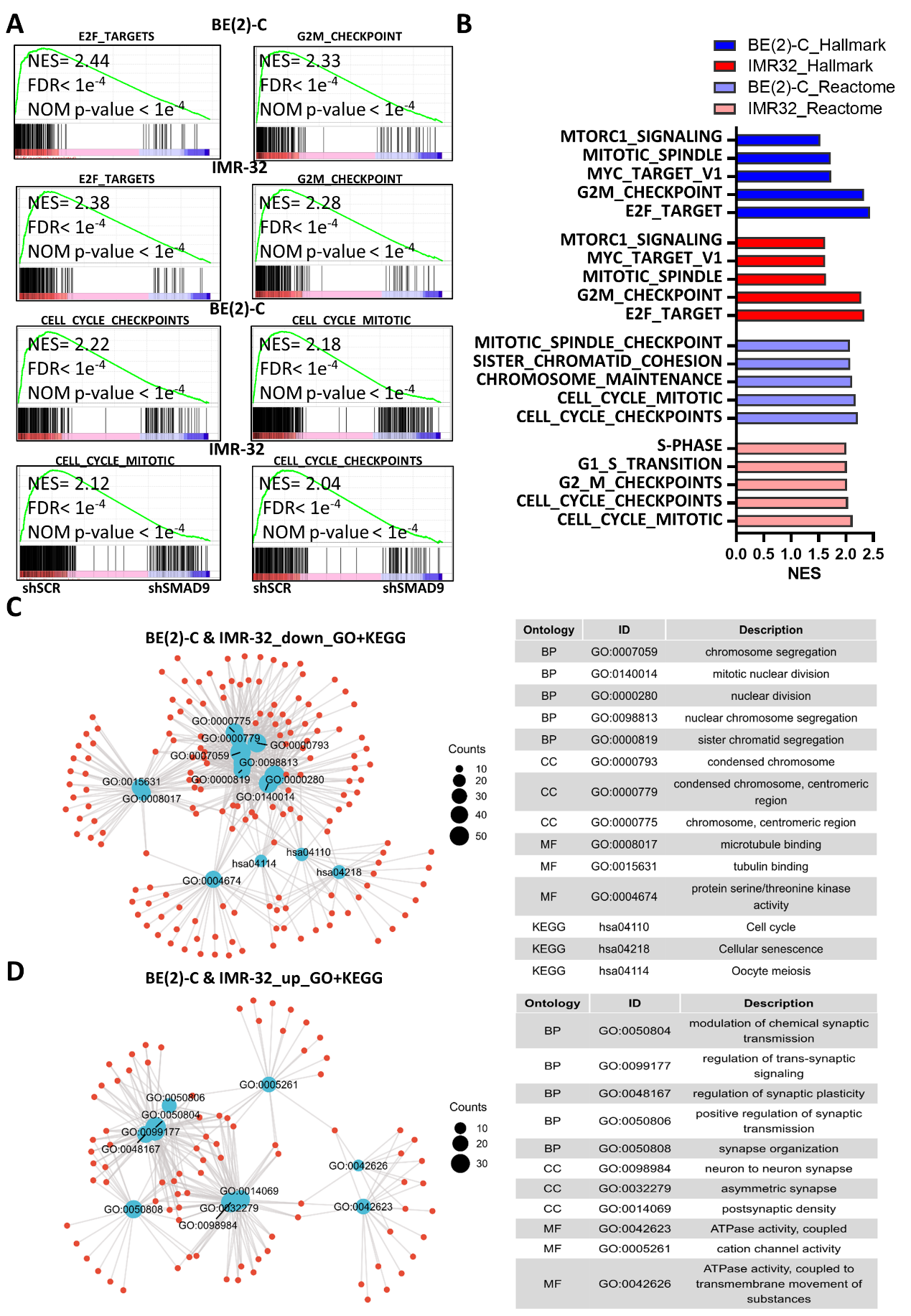
***

**Figure S8. Transcriptome changes in response to SMAD9 knockdown.**

***(A, B)*** *The representative “Hallmark” (upper panel) and “Reactome” (lower panel) GSEA items in BE(2)-C and IMR-32 cells with FDR and NOM p values.* ***(C, D)*** *Maps showing the top 3 GO/KEGG terms based on the 784 overlapping downregulated genes (C) and 570 overlapping upregulated genes (D).*

*BP: biological process; CC: cell component; FDR: false discovery rate; GSEA: gene set enrichment analysis; KEGG: Kyoto Encyclopedia of Genes and Genomes; MF: molecular function; NOM: normalized.*

**
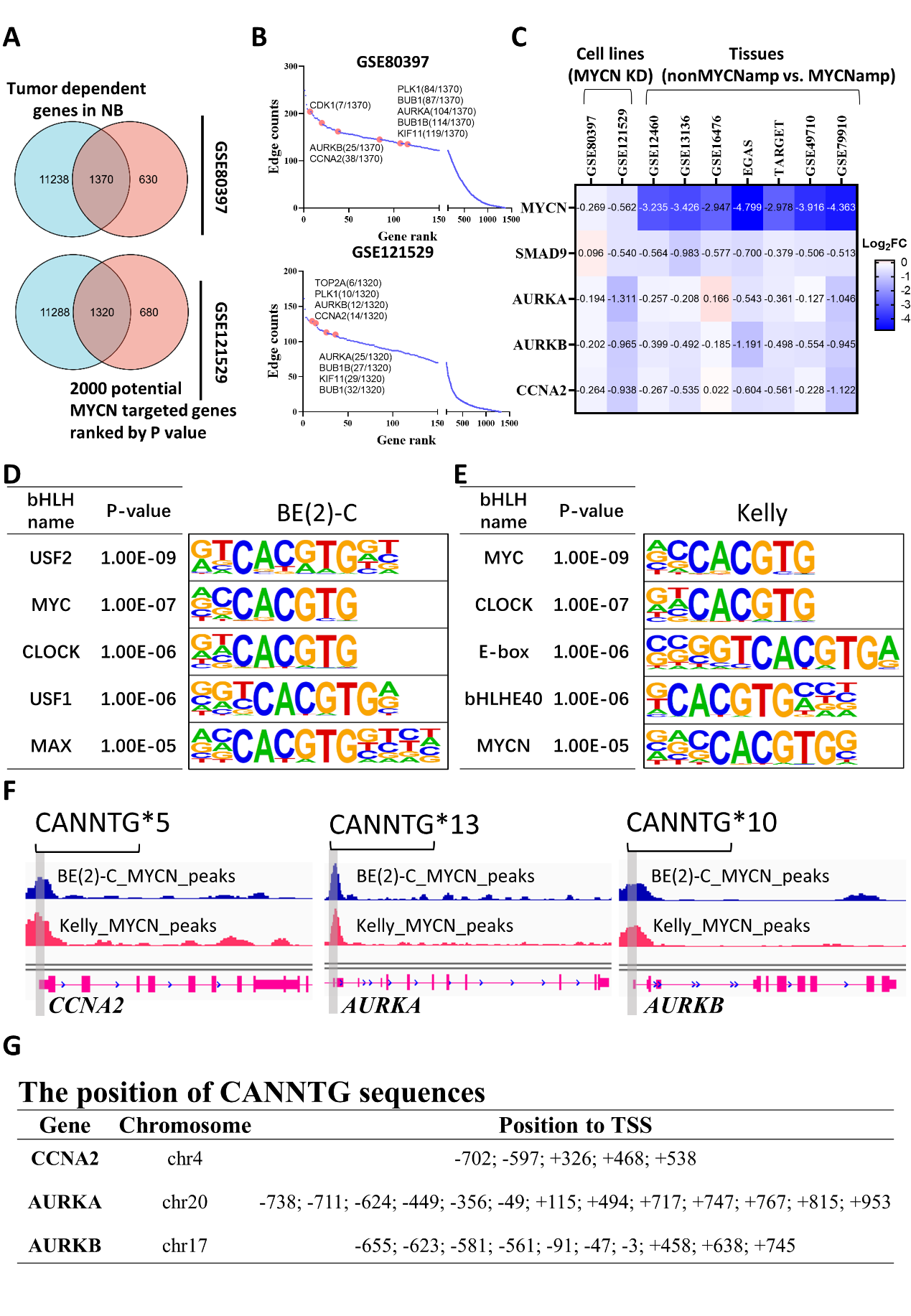
**

**Figure S9. Transcriptome changes in response to MYCN knockdown and binding patterns of E-box sequences.**

***(A)*** *Venn diagram showing the overlapping downregulated genes between tumor dependent genes (the genes in NB with dependency score < 0 in the DepMap portal) and DEGs induced by MYCN knockdown extracted from GSE80397 and GSE121529.* ***(B)*** *Genes ranked by edge counts based on the PPI network and the top 10 hub genes in SMAD9-high cells are highlighted.* ***(C)*** *Heatmap showing changes in the expression of some hub genes in NB cells after MYCN knockdown and NB tumors in non-MYCN-amp tissues.* ***(D, E)*** *The E-box binding motifs in the anti-MYCN ChIP-seq results.* ***(F, G)*** *The E-box sequences (CANNTG) in the promoter of the cell cycle hub genes (F), and the specific position of the CANNTG sequences relative to TSS (G). The ChIP-seq data were extracted from GSE94822.*

*ChIP: chromatin immunoprecipitation; ChIP-seq: ChIP sequencing; DEGs: differentially expressed genes; KD: knockdown; MYCN-amp: MYCN amplification; NB: neuroblastoma; non-MYCN-amp: non-MYCN amplification; TSS: transcription start site.*
